# Extrapolation of lung pharmacokinetics of antitubercular drugs from preclinical species to humans using PBPK modelling

**DOI:** 10.1093/jac/dkae109

**Published:** 2024-04-10

**Authors:** Evangelos Karakitsios, Aristides Dokoumetzidis

**Affiliations:** Department of Pharmacy, University of Athens, Panepistimiopolis Zografou, 15784 Athens, Greece; Pharma-Informatics Unit, Athena Research Center, Artemidos 6 & Epidavrou, 15125 Marousi, Greece; Institute for Applied Computing “Mauro Picone”, National Research Council (CNR), Via dei Taurini 19, 00185 Rome, Italy; Department of Pharmacy, University of Athens, Panepistimiopolis Zografou, 15784 Athens, Greece; Pharma-Informatics Unit, Athena Research Center, Artemidos 6 & Epidavrou, 15125 Marousi, Greece; Institute for Applied Computing “Mauro Picone”, National Research Council (CNR), Via dei Taurini 19, 00185 Rome, Italy

## Abstract

**Objectives:**

To develop physiologically based pharmacokinetic (PBPK) models for widely used anti-TB drugs, namely rifampicin, pyrazinamide, isoniazid, ethambutol and moxifloxacin lung pharmacokinetics (PK)—regarding both healthy and TB-infected tissue (cellular lesion and caseum)—in preclinical species and to extrapolate to humans.

**Methods:**

Empirical models were used for the plasma PK of each species, which were connected to multicompartment permeability-limited lung models within a middle-out PBPK approach with an appropriate physiological parameterization that was scalable across species. Lung’s extracellular water (EW) was assumed to be the linking component between healthy and infected tissue, while passive diffusion was assumed for the drug transferring between cellular lesion and caseum.

**Results:**

In rabbits, optimized unbound fractions in intracellular water of rifampicin, moxifloxacin and ethambutol were 0.015, 0.056 and 0.08, respectively, while the optimized unbound fractions in EW of pyrazinamide and isoniazid in mice were 0.25 and 0.17, respectively. In humans, all mean extrapolated daily AUC and *C*_max_ values of various lung regions were within 2-fold of the observed ones. Unbound concentrations in the caseum were lower than unbound plasma concentrations for both rifampicin and moxifloxacin. For rifampicin, unbound concentrations in cellular rim are slightly lower, while for moxifloxacin they are significantly higher than unbound plasma concentrations.

**Conclusions:**

The developed PBPK approach was able to extrapolate lung PK from preclinical species to humans and to predict unbound concentrations in the various TB-infected regions, unlike empirical lung models. We found that plasma free drug PK is not always a good surrogate for TB-infected tissue unbound PK.

## Introduction

TB remains a lethal disease and a major cause of death worldwide. In 2021, an estimated 10.6 million people developed TB, and 1.6 million patients died. The disease typically affects the lungs (pulmonary TB) but can affect other sites as well.^1^ Even though TB regimens for drug-susceptible strains have high efficacy in the clinical trials, they remain unsatisfyingly effective for MDR and XDR cases.^2^ Furthermore, the complex pathology of TB in the lung tissue is one of the main difficulties in improving TB regimens. During the first stages of the disease, highly vascularized cellular granulomas are formed, which are rich in immune cells. At this stage bacilli exist, extracellularly and intracellularly in foamy macrophages. As the disease progresses, the granulomas begin to necrotize from the centre outwards and only the cellular rim remains well vascularized, while a necrotic caseum in the centre is formed. At this step, bacilli reside extracellularly in the caseum and intracellularly in various immune cells of the cellular rim. Finally, when an expanding granuloma meets an airway, cavities are formed.^3^ All these heterogenous lesions in pulmonary TB lead to limited drug access to the site of infection and it has been shown that drug pharmacokinetics (PK) over time, in the various lung lesions, poorly correlate with the drug PK in blood.^4,5^ It is also noted that in these diverse microenvironments of the lung, drug concentrations cannot be readily measured.^3^

Clinically efficacious doses for antitubercular drugs are currently being forecast based largely on plasma free drug levels. However, as mentioned, the formed lesions in TB can alter the PK of drugs. Physiologically based pharmacokinetic (PBPK) modelling offers the potential to make predictions of drug levels in the various lung compartments, linking the lung PK with the blood/plasma PK.^6^ Additionally, an accurate physiologically relevant parameterization of the lung offers the possibility of extrapolation across species, within a framework that includes uncertainty in a quantifiable way.^7^ Utilizing the good *in vivo* characterization of the drug PK in the infected lung through PBPK modelling, the well-characterized *in vitro* or preclinical efficacy, through mechanistic pharmacodynamic (PD) relationships, could ultimately be translated to clinical efficacy by *in silico* clinical trials.

In our current analysis the objective was 2-fold. The first objective was to extrapolate the lung PK profile, regarding both healthy/uninvolved lung and infected tissue (cellular rim and caseum), of currently used anti-TB drugs, from preclinical species to humans, to investigate whether plasma drug exposure is a good surrogate measure for lung exposure. The second objective was to provide the unbound drug concentrations in each of these lung compartments since experimental measurements reflect total drug in homogenates and not unbound drug in separate intracellular/extracellular spaces, while it is the unbound fraction of drug driving the PD effect.

## Methods

### In vivo data availability

Literature was searched for lung PK profiles over time of currently used anti-TB drugs in humans and in at least one preclinical species. The search was conducted in PubMed using keywords such as ‘tuberculosis’, ‘lung pharmacokinetics’ and ‘homogenate concentrations’. It is noted that almost all plasma PK profiles were described by empirical models that were publicly available in the articles we eventually found. Whenever plasma PK profiles were not available, the raw data were utilized to develop empirical plasma PK models. Regarding lung concentrations, only dissected lung tissue homogenate (from various regions of the TB lung) and alveolar cell concentrations were used to quantitatively describe the lung distribution over time, while qualitative or semi-quantitative approaches (such as matrix-assisted laser desorption/ionization MS imaging) were ignored.^8^ Also, whenever lung concentrations of a specific region of the lung over time were not directly available, they were captured from the respective figures using digitizer software.

Finally, through our search, plasma and lung PK profiles, concerning both healthy and infected lung, were found for pyrazinamide, rifampicin, ethambutol and isoniazid in mice, for rifampicin, ethambutol and moxifloxacin in rabbits and for all the previously mentioned five drugs in humans, as summarized in Table 1.^9–18^ These data were utilized to develop lung PBPK models in the PK software Monolix Suite 2021R1 (Simulations Plus, Lancaster, CA, USA).

**Table 1.** All available literature data concerning lung PK of typical anti-TB drugs (rifampicin, ethambutol, moxifloxacin, pyrazinamide and isoniazid) that were found in at least two species (mouse, rabbit or human)

Drug	Site	Source		Target	
		Species	Dose	Species	Dose
Rifampicin	Uninvolved lung	Mouse^10^	10 mg/kg (PO, SD)	Human^16,18^	600 mg (PO, SD)
	Uninvolved lung, cellular lesion, caseum	Rabbit^11^	10 mg/kg (IV, SD/MD)		600 mg (PO, SD)
Ethambutol	Uninvolved lung	Mouse^10^	100 mg/kg (PO, SD)		15 mg/kg (PO, MD)
	Uninvolved lung	Rabbit^12^	100 mg/kg (PO, SD/MD)		15 mg/kg (PO, MD)
Moxifloxacin	Uninvolved lung, cellular lesion, caseum	Rabbit^13,15^	100 mg/kg (PO, SD)		400 mg (PO, SD)
Pyrazinamide	Uninvolved lung, cellular lesion	Mouse^9^	150 mg/kg (PO, SD)		1500 mg (PO, SD)
Isoniazid	Uninvolved lung	Mouse^10^	5 mg/kg (PO, SD)		300 mg (PO, SD)

PO, per os; SD, single dose; MD, multiple doses. The lung distribution over time concerns either the healthy/uninvolved tissue or the infected tissue (cellular lesion or caseum). For each drug, the extrapolation of lung PK was performed from either mice or rabbits to humans for healthy lung, cellular lesion or caseum, based on availability of literature data, as listed.

### Extrapolation strategy for lung PK

Preclinical lung PK of several anti-TB drugs was extrapolated to humans, using a middle-out approach. At first, empirical plasma PK models were either utilized from literature or built for both preclinical species and humans. Next, middle-out PBPK models were developed instead of whole-body PBPK models to lower the uncertainty of predicted lung PK profiles. Whole-body PBPK models often do not accurately predict plasma concentrations over time and this can propagate to an uncertain lung PK profile.

Multicompartment permeability-limited lung PBPK models were built, taking into consideration the physiology of each species, as well as the drug-related parameters of each compound. The central compartment of the empirical plasma model was connected to a multicompartment lung, which is composed of the pulmonary blood (PB), the extracellular water (EW) and intracellular water (IW). An instant equilibrium was assumed between PB and EW. Also, there was permeability-limited transfer between EW and IW. Note that there is no mass balance between the empirical PK model and the lung model since the contribution of the lung PK is already taken into account in the kinetics of the empirical plasma PK model.

Regarding the distribution of drugs in healthy/uninvolved lung, the studied drugs were classified into two main categories: compounds that are moderate to strong bases, which have at least one basic pK_a_ ≥ 7 (rifampicin, moxifloxacin and ethambutol); and weak bases, which are molecules with no basic pK_a_ ≥ 7 (pyrazinamide and isoniazid). A basic assumption of our model is that strong to moderate bases will predominantly bind to the acidic phospholipids of IW, while weak bases will mainly bind to the albumin of EW. This is in line with the widely used mechanistic model by Rodgers and Rowland.^19–21^

In Table 2 the parameters of the permeability-limited lung PBPK models are depicted. These parameters include drug-related parameters, species-related parameters, parameters different for each drug and each species, as well as some parameters that were either fitted using lung PK profiles in preclinical species or calculated based on basic assumptions.

**Table 2.** Lung permeability-limited PBPK parameters of healthy/uninvolved lung

Model parameter	Type
Molecular weight	Drug-related parameters (independent of species)
pKa value	
Lipophilicity—calculated logP value	
Blood flow rate	Species-related parameters (independent of drug)
Volume of lung PB	
Volume of EW	
Volume of IW	
Cellular surface area	
Surface area of capillaries	
pH values in the three lung compartments (PB, EW and IW)	
Fraction unbound in plasma (*fu*_pls_)	Different for each drug and each species
Blood to plasma ratio (B/P)	
Fraction unbound in EW (*fu*_EW_)	Calculated based on Rodgers & Rowland theory or fitted using preclinical lung PK profiles
Fraction unbound in IW (*fu*_IW_)	
Permeability of lung cells	

**Figure 1.**
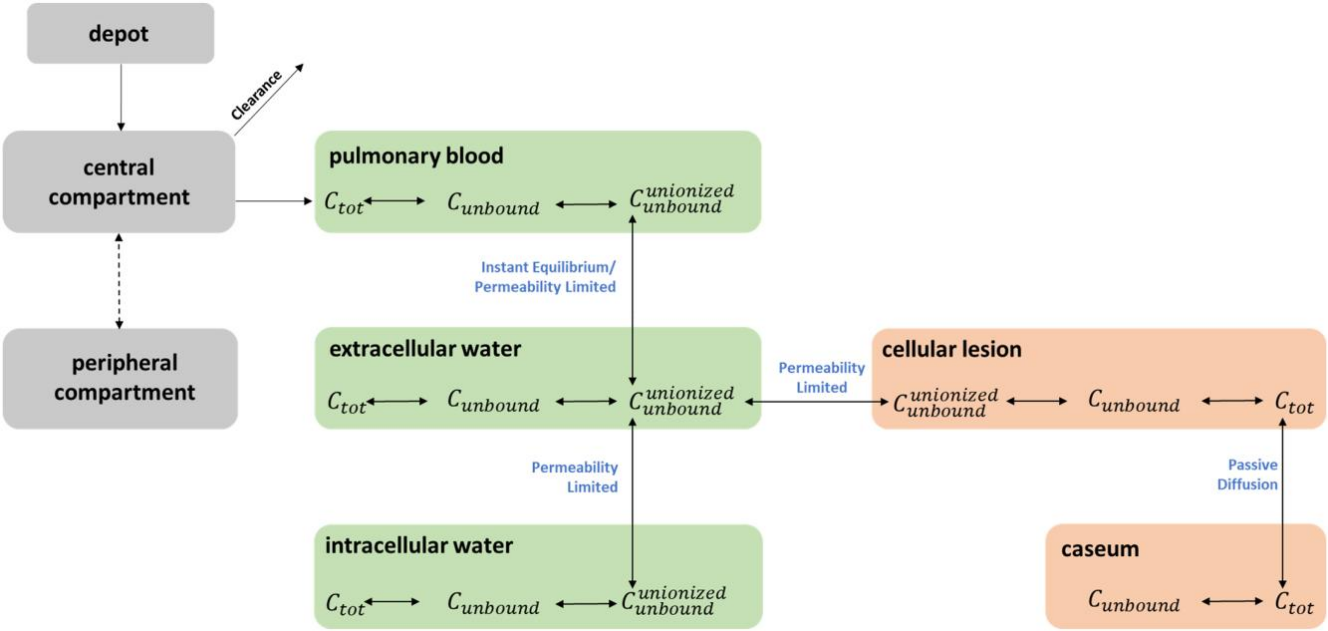
Diagram of our developed middle-out multicompartment permeability-limited lung PBPK models. Empirical models were used to describe the plasma PK: a depot compartment is used when the dose is administered orally, the central compartment describes either the blood or plasma PK of the drug that is cleared from the central compartment, while peripheral compartments are used when necessary. The central compartment is connected to a multicompartment lung, which is composed of the PB, the EW and the IW. Also, an instant equilibrium is assumed between the PB and the EW, while there is permeability-limited transfer between the EW and the IW. On the right, the diagram is extended to include the cellular lesion (macrophages) and the caseum as well. The EW is the linking component between the healthy and the infected tissue. Permeability-limited transfer is assumed between the EW and the cellular lesion. Moreover, passive diffusion is assumed for the drug transferring between the cellular lesion and the caseum. This figure appears in colour in the online version of *JAC* and in black and white in the print version of *JAC*.

For the infected tissue, the linking component between healthy/uninvolved and TB-infected lung is EW. The drug in EW can permeate the cellular membrane of either the healthy/uninvolved lung cells or macrophages, which form the cellular lesions in our model. Finally, the drugs are assumed to passively diffuse from the cellular lesion to the caseum.^22,23^ The final middle-out multicompartment permeability-limited lung PBPK model is depicted in Figure 1.

Key distribution parameters, regarding both healthy/uninvolved lung and infected tissue, were estimated utilizing the respective preclinical *in vivo* lung PK profiles. These parameters involved affinity constants obtained through fraction unbound (e.g. affinity constant for lung acidic phospholipids in the case of moderate to strong bases), lung permeability values regarding healthy/uninvolved tissue, as well as rates and ratios of distribution concerning the infected lung tissue. *In vitro* values, such as pH values of cellular lesions, intracellular to extracellular ratios for macrophages, and human surrogate fraction unbound values in caseum were also utilized.^9,14,23–25^ Finally, the lung PK profiles were extrapolated to humans by keeping the values of these distribution parameters constant across species and altering appropriately the physiology of each species.

The predicted/extrapolated lung PK profiles to humans were compared with the respective observed biopsy data from literature. Note that data used in the preclinical models as well as data used to validate the human model concern total drug concentrations as measured experimentally in homogenate tissue, which could be different by orders of magnitude compared with unbound drug concentrations. In order to calculate the total drug concentration in homogenate, the intracellular, extracellular and vascular concentrations, as well as their corresponding volumes, are taken into account. Through the PBPK parameterization of the model, the unbound drug concentrations in humans for each lung compartment can be predicted, which is one of the strengths of the current approach.

It is also noted that since P-gp is an efflux transporter on the apical membrane of the pulmonary epithelial and vascular endothelial cells, a time-independent distribution of moxifloxacin between PB and EW cannot be considered valid.^26,27^ Hence, permeability-limited transfer was also assumed between these two compartments. This highlights the vital importance of taking into consideration each compound’s special characteristics for lung PK extrapolation.

The estimation of parameters was performed in Monolix, which uses the stochastic approximation expectation-maximization algorithm.^28^ Moreover, very recently, a local sensitivity analysis was performed on a minimal PBPK model for antitubercular drugs, listing AUC and *C*_max_ as the most informative measures in terms of model response to perturbations. The authors concluded that both plasma and lung PK profiles were well summarized by AUC and *C*_max_ values.^29^ Hence, in our analysis, these measures were used as metrics of model performance for extrapolation purposes together with visual inspection of concentration–time profiles.

Our extrapolation strategy is presented in detail in the Supplementary Methods (Method Development, available as Supplementary data at *JAC* Online), with all the assumptions that were made (Table S1) and the respective differential equations and all the parameters (Tables S2–S8) that were used in the PBPK models. Moreover, a list of datasets (both *in vitro* and *in vivo*) required for this work is presented, detailing which datasets were used in the estimation and which in the extrapolation step (Table S9).

Regarding the evaluation of the extrapolation step to humans, it is noted that the raw data in humans for all drugs (except for ethambutol), for both the healthy and infected tissue, were not available and it was very difficult to digitize them.^17^ However, in that study, empirical PK models were reported that characterize quantitatively this data.^17^ Hence, to compare the PBPK models with the available observations visually, simulations with the reported empirical equations were performed and also AUC and *C*_max_ values could be calculated. Consequently, all extrapolated PK profiles in humans represent a typical mean patient.

Concerning the extrapolation of human PK of rifampicin from rabbit data, the empirical plasma PK in rabbits after an IV infusion that has been developed by Rifat *et al.*^11^ was utilized in our model. It is noted that the observed data that were used for the fitting of the cellular lesion PK were both the cellular and cavity wall lung data. These data were grouped together to increase the number of datapoints (five datapoints in total after single and multiple dosing). Furthermore, regarding extrapolation of rifampicin from mice, initially an empirical model from the literature was utilized to describe the blood PK of rifampicin in mice after an oral administration of 10 mg/kg, which is the human equivalent dose in mice.^30,31^

The same approach was followed for all other drugs for their lung PK extrapolation from preclinical species to humans, taking always into consideration their human equivalent doses in animals. It is also noted that in order to derive the empirical plasma PK of ethambutol in humans, we digitized and utilized the mean raw serum concentrations after an oral administration of 25 mg/kg of the drug in healthy individuals.^16^

## Results

### Extrapolation of total lung concentrations

The optimized parameters of the PBPK models for all drugs, utilizing preclinical lung PK profiles, are shown in Table 3. The fitted lung PK profiles, of both healthy/uninvolved and infected tissue, are shown in Figures S1 and S2 for rifampicin, Figure S3 for moxifloxacin, Figure S5 and S6 for ethambutol, Figure S7 for pyrazinamide and Figure S8 for isoniazid. Also, the fitting of plasma PK of ethambutol in rabbits is depicted in Figure S4, with the respective parameter estimates in Table S10.

**Table 3.** Estimated lung PBPK parameters for rifampicin (RIF), moxifloxacin (MXF), ethambutol (ETH), isoniazid (INH) and pyrazinamide (PZA) using preclinical *in vivo* data^a,b^

Parameter	RIF	MXF	ETH	INH	PZA
Cellular permeability (cm/h) (RSE, %)	Rabbits 3.22E+4 (31.1)	Rabbits 9.9 (0.10)	Rabbits 0.23 (18.8)	Mice 3.7E−4 (19.6)	Mice 2.1E−4 (63.9)
	Mice 1.45E+6 (61.0)		Mice 0.34 (13.6)		
Capillary permeability (cm/h) (RSE, %)	Rabbits/mice —	Rabbits 6.0 (19.7)	Rabbits/mice —	Mice —	Mice —
Fraction unbound in IW (*fu*_IW_)^c^ (RSE, %)	Rabbits 0.015 (8.6)	Rabbits 0.056 (20.4)	Rabbits 0.08 (10.5)	Mice 0.97	Mice 0.95
	Mice 8.4E−3 (9.7)		Mice 0.056 (12.4)		
Fraction unbound in EW (*fu*_EW_)^d^ (RSE, %)	Rabbits/Mice 1	Rabbits 1	Mice/Rabbits 1	Mice 0.17 (3.83)	Mice 0.25 (6.78)
Rate of distribution in cellular lesion (L/h) (RSE, %)	Rabbits 1.58E+7 (15.0)	Rabbits 2.19E+4 (34.5)	—	—	Mice 72.5 (54.4)
Product of fraction non-ionized × fraction unbound in cellular lesion (RSE, %)	Rabbits 1.6E−8 (12.2)	Rabbits 4.7E−5 (22.7)	—	—	Mice 0.53 (3.04)
Rate of distribution in caseum (L/h) (RSE, %)	Rabbits 0.22 (23.8)	Rabbits 9.64 (135)	—	—	—
Ratio of distribution in caseum (RSE, %)	Rabbits 1.5 (32.9)	Rabbits 0.49 (14.0)	—	—	—

^a^In cases where two preclinical species were used for the extrapolation, the cells are split in two rows to determine the value of one parameter for each case.

^b^Relative standard error (RSE, %) describes the precision of the parameters generated by Monolix.

^c^ *fu*_IW_ was optimized for the moderate to strong bases RIF, MXF and ETH, while for the weak bases INH and PZA it was predicted assuming that the affinity constant for acidic phospholipids is 0.

^d^ *fu*_EW_ was assumed to be 1 for the moderate to strong bases RIF, MXF and ETH, while for the weak bases INH and PZA it was optimized.

**Figure 2.**
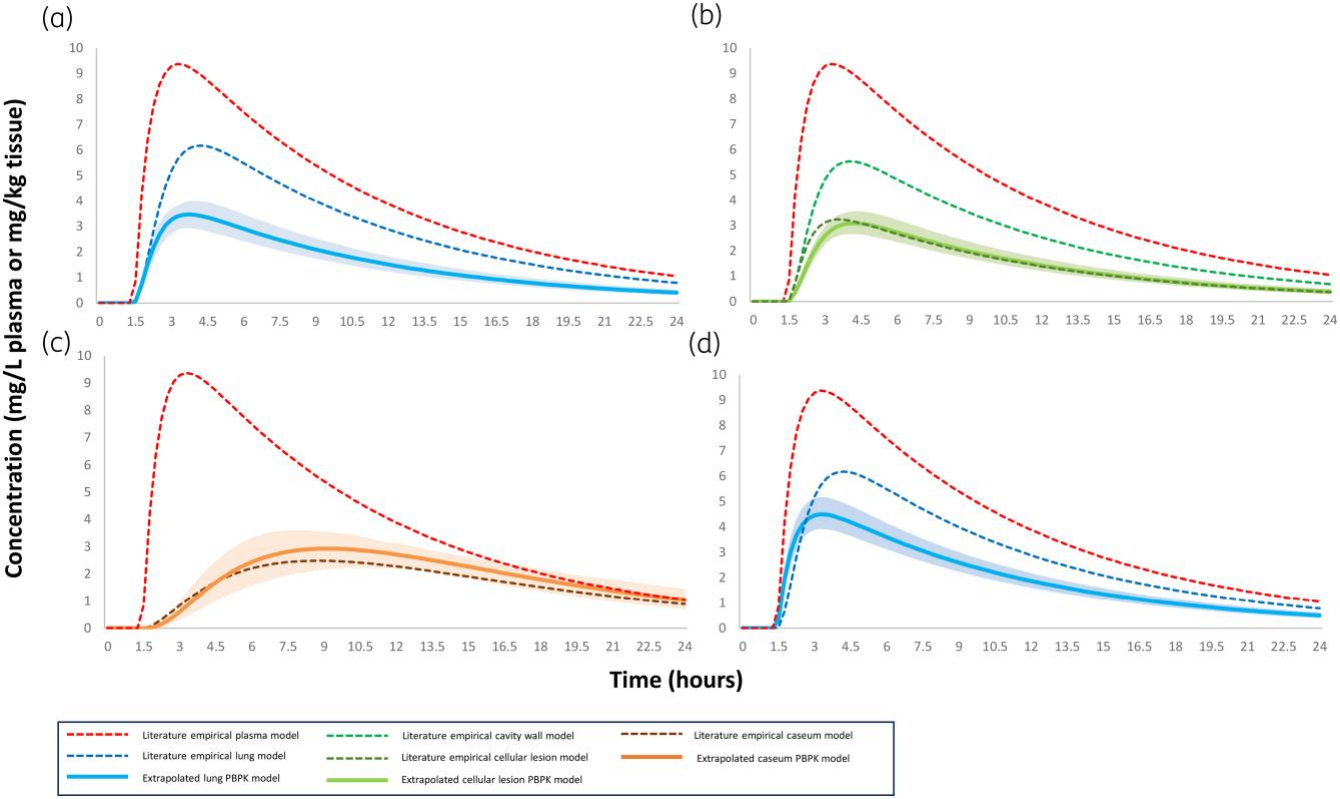
Diagram of extrapolated lung PK profile of rifampicin in humans. The dashed red line represents the plasma PK of a typical mean TB patient after an oral dose of 600 mg of rifampicin, while the dashed blue, green and brown lines represent the empirical literature healthy/uninvolved lung (a and d), cellular lesion or cavity wall (b) and cavity caseum (c) PK profiles of humans, respectively.^17^ The solid light blue, green and brown lines depict our extrapolated lung PBPK profile of healthy lung, cellular lesion and cavity caseum, respectively, in TB patients. In (a), (b) and (c), the extrapolation was performed from rabbits, while in (d) the extrapolation was performed from mice. The shaded areas refer to the uncertainty of predictions in humans, taking into consideration the relevant relative standard errors of the estimated parameters in Monolix. This figure appears in colour in the online version of *JAC* and in black and white in the print version of *JAC*.

The respective extrapolated lung PK profiles of rifampicin in humans, representing a mean typical patient, are depicted in Figure 2. Additionally, extrapolated PK profiles of all other drugs in humans, representing a typical mean patient, are depicted in Figure S9 for moxifloxacin, Figure S12 for ethambutol, Figure S13 for pyrazinamide and Figure S14 for isoniazid. Moreover, sensitivity analysis was performed on moxifloxacin’s capillary permeability (Figure S10) and, also, plasma PK of ethambutol were derived for humans (Table S11, Figure S11).

**Figure 3.**
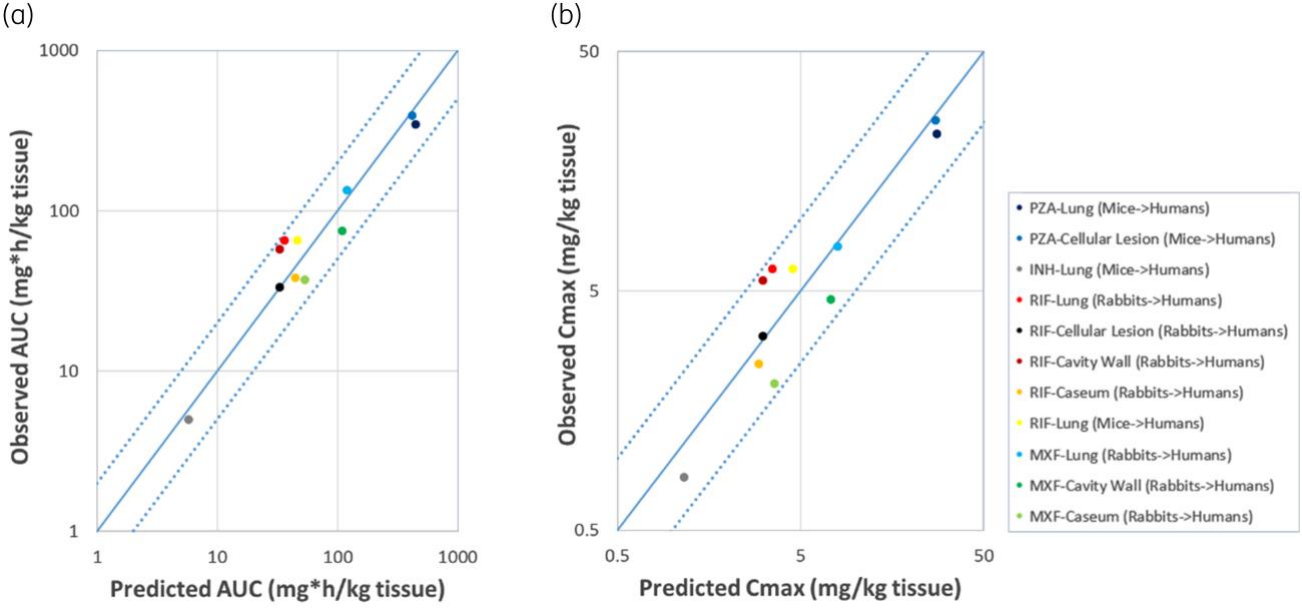
Mean observed versus extrapolated/predicted daily AUC (a) and *C*_max_ (b) after a single-dose daily administration of isoniazid (INH), pyrazinamide (PZA), moxifloxacin (MXF) and rifampicin (RIF). To the right-hand side, next to the drug’s name, the specific site of the lung is indicated and in parentheses the species from which the extrapolation was performed is shown, based on the availability of literature data (each drug and site of lung is indicated with a different colour). This figure appears in colour in the online version of *JAC* and in black and white in the print version of *JAC*.

Furthermore, the respective observed and predicted daily AUC, *C*_max_ and *T*_max_ values after the oral administration of all five drugs are shown in Table 4. Also, in Figure 3, the observed versus predicted daily AUC and *C*_max_ are depicted. As shown in Figure 3, all predictions were within 2-fold compared with the observed values, meeting the reference model-fidelity criterion.^32^

**Table 4.** Observed versus predicted daily AUC, *C*_max_ and *T*_max_ values of lung tissue in humans in different sites, extrapolated from rabbits and mice

Site	Source species	AUC_24_ (mg·h/kg)		*C*_max_ (mg/kg)		*T*_max_ (h)	
		Observed	Predicted	Observed	Predicted	Observed	Predicted
Rifampicin							
Uninvolved	Rabbit	65.56	35.7	6.19	3.48	4.25	3.75
Cellular lesion	Rabbit	33.06	32.49	3.24	3.08	3.5	4.25
Cavity wall	Rabbit	58.08	32.49	5.54	3.08	4	4.25
Caseum	Rabbit	38.39	44.02	2.49	2.93	8.75	9.25
Uninvolved	Mouse	65.56	45.73	6.19	4.5	4.25	3.25
Moxifloxacin							
Uninvolved	Rabbit	135.18	118	7.72	7.92	7.75	3.75
Cavity wall	Rabbit	75.07	108.54	4.63	7.29	5.75	3.75
Caseum	Rabbit	37.29	53.17	2.06	3.58	9	4
Pyrazinamide							
Uninvolved	Mouse	346.93	441.52	22.77	27.58	5	6
Cellular lesion	Mouse	395.91	415.05	25.88	27.14	4	4.25
Isoniazid							
Uninvolved	Mouse	5.03	5.73	0.84	1.15	3	2

Doses were single oral 600, 400, 1500 and 300 mg, for rifampicin, moxifloxacin, pyrazinamide and isoniazid, respectively.

### Prediction of free-drug lung concentrations

**Figure 4.**
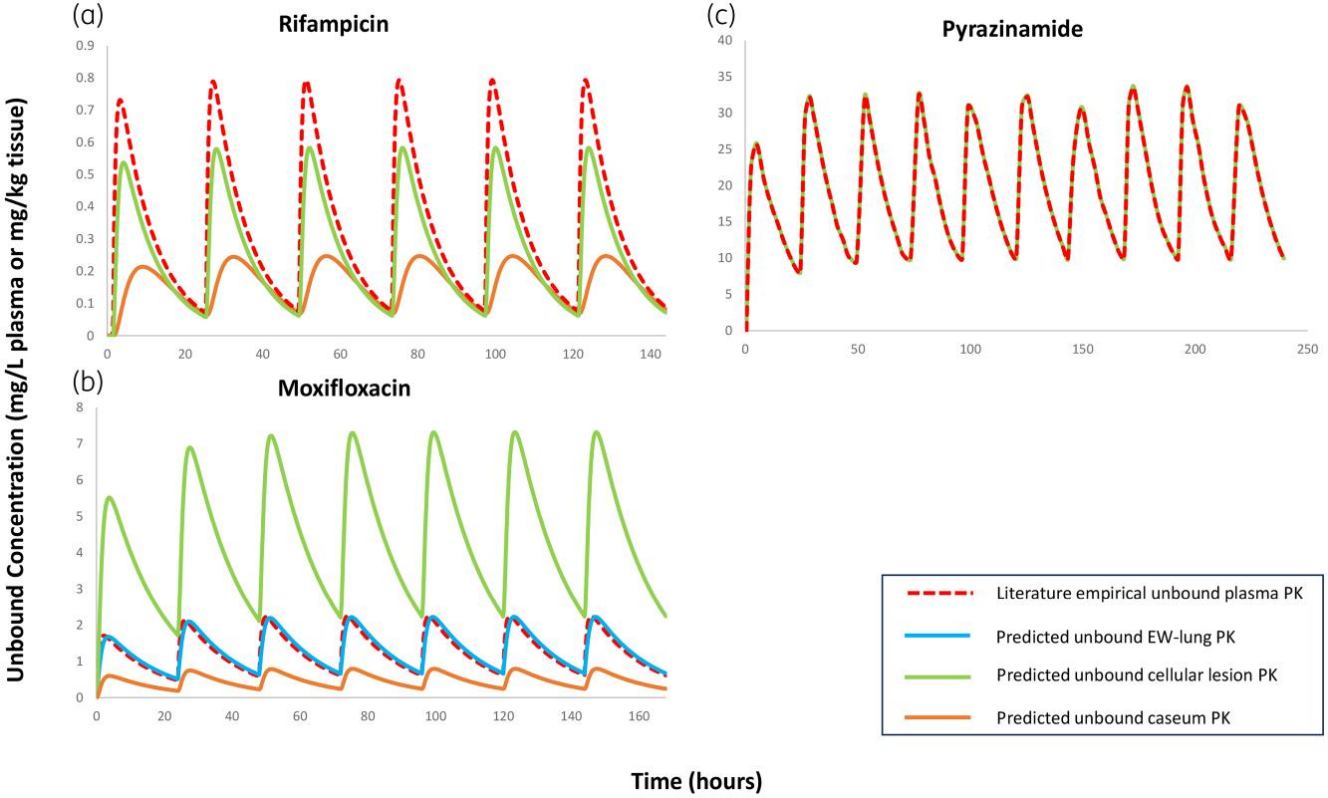
Diagram of the predicted free lung PK profiles in humans in plasma, as well as in the compartments where bacteria reside (EW, cellular lesions and caseum) for rifampicin, moxifloxacin and pyrazinamide. (a) Rifampicin after multiple oral doses of 600 mg; (b) moxifloxacin after multiple oral doses of 400 mg; (c) pyrazinamide after multiple oral doses of 1500 mg. In all graphs, the red dashed line represents the unbound plasma concentration in humans, while the solid blue, green and brown lines refer to the unbound concentrations in EW, cellular lesions and caseum, respectively. For rifampicin and pyrazinamide, unbound EW concentrations over time (blue solid lines) were not plotted to improve readability, since they were identical to the unbound plasma ones (dashed red lines). This figure appears in colour in the online version of *JAC* and in black and white in the print version of *JAC*.

While the data utilized concerned total drug concentrations in homogenates, the PBPK parametrization used allows the prediction of free-drug PK profiles in the separate lung compartments where bacteria reside (EW, cellular lesion and caseum). The predicted unbound fractions are shown in Table S12, while the free-drug PK profiles are depicted in Figure 4. As shown in Figure 4, for moxifloxacin there is only a slight deviation between the unbound concentrations in EW and plasma (Figure 4b), which can be attributed to moxifloxacin being a P-gp substrate.

Moreover, unbound concentrations in the caseum are lower compared with unbound plasma concentrations for both rifampicin and moxifloxacin (Figure 4a and b). Furthermore, for rifampicin, unbound concentrations over time in cellular rim are slightly lower compared with unbound plasma concentrations (Figure 4a). On the contrary, for moxifloxacin, unbound cellular concentrations are significantly higher (Figure 4b) than unbound plasma concentrations, while for pyrazinamide, unbound concentrations in cellular lesions over time are almost identical to unbound plasma concentrations (Figure 4c).

It is noteworthy that rifampicin and moxifloxacin exhibit different unbound cellular lesion PK profiles compared with unbound plasma PK profiles, although they have quite similar intracellular to extracellular ratios for macrophages (4.35 and 4.4, respectively).^14,25^ This can be explained by their different ionization in these compartments. At steady state, which is achieved after a few days for these ampholytes, unbound unionized concentrations in EW and cellular lesion will be equal. Therefore, at steady state, the ratio of unbound concentrations in EW and cellular lesion will be equal to the ratio of the non-ionized fractions in these two compartments. For rifampicin this ratio is 1.3, while for moxifloxacin it is only 0.3 since rifampicin has a very lower acidic pKa (pKa_1_ = 1.7) compared with moxifloxacin (pKa_1_ = 6.25).

## Discussion

The presented analysis makes several assumptions and also has some limitations. Only mean lung data were utilized in preclinical species since mostly only these were available (mean digitized literature values from three animals). Furthermore, in TB patients exclusively, mean lung data could be utilized. The variability associated with the lung concentration data, which was often sparse and very likely included within-subject and inter-subject variability, which may be caused by formulation differences as well as baseline covariates, along with inter-laboratory variability, could be lowered with the utilization of the specific raw data.

It is also noteworthy that for all drugs human-equivalent doses, in terms of PK (or available doses that best matched human equivalent doses) were used in preclinical species since in certain cases, the optimized parameters found were significantly different among different doses. Consequently, for future extrapolation of novel drugs’ lung PK from preclinical species to humans it is always advisable to use the intended human-equivalent dose in animal experiments.

In addition, the highest relative standard errors, as shown in Table 3, were found for the permeability of lung cells (for rifampicin and pyrazinamide) and for the rate of distribution of the caseum (for moxifloxacin). Measured *in vitro* permeability values could be used for new molecules, instead of optimized ones, for healthy lung cells. For rifampicin, the apparent permeability value in Calu-3 cells (basolateral to apical direction) is 60.84 × 10^−4^ cm/h, which divided by the unionized fraction of rifampicin at pH 7.4 (4.7 × 10^−7^) gives a value equal to 12.9 × 10^3^ cm/h.^33^ The predicted lung PK profile for rifampicin in humans using this *in vitro* permeability value produced a *T*_max_ value (4.50 h) closer to the observed one (4.25 h) compared with the *T*_max_ values obtained with the optimized permeability values in preclinical species.

It is also noted that in order to extrapolate the caseum PK more accurately from rabbits to humans, lung cavities similar (diameter of 4.2 mm) to those of TB patients should be developed in animals.^11^ This experimental procedure led to better extrapolation in our analysis for rifampicin (Figure 2c) compared with moxifloxacin (Figure S9c). Additionally, concerning the extrapolation step of the infected tissue, since physiology data were not available, we used a semi-PBPK approach, keeping the rates of distribution (cellular lesion and caseum) and the ratio of distribution (caseum) the same among species.

Imaging data in the future could be helpful to shed light on the infected tissue’s physiology, such as shape of necrotic granulomas, and hence use a more mechanistic description of the biological process that refers to the passive diffusion of the drugs from the cellular rim to the caseum and, finally, utilize an entirely physiologically based approach for the extrapolation. Nevertheless, PBPK predictions were within a reasonable 2-fold difference from literature-observed concentrations in humans. However, this difference could be lowered even further by the collection of much more appropriate data.

Furthermore, according to our model, moxifloxacin’s distribution into the whole lung compartment will be determined by the lower capillary permeability and not lung cellular permeability (see Table 3). Unfortunately, *in vitro* data concerning capillary permeability of moxifloxacin were not available. However, the following arguments point to the capillary permeability (and not the permeability of lung cells) being the limiting factor for the permeation of moxifloxacin into the lung: (i) moxifloxacin’s transport through the Calu-3 cells is assumed to be in part active at the luminal side and mostly passive at the basolateral side; (ii) its apparent permeability (apical to basolateral direction) across monolayers of WT Madin–Darby canine kidney cells is really low (5.7 × 10^−6^ cm/s); and (iii) P-gp seems to be expressed abundantly in the luminal membrane of pulmonary capillary endothelial cells.^34–36^ This highlights the importance of including P-gp-mediated transport through pulmonary endothelial cells when investigating the lung distribution of a P-gp substrate.

In this work, middle-out multicompartment permeability-limited lung PBPK models of commonly used anti-TB drugs were developed for preclinical species based on available literature data, with appropriate physiological parameterization. The multicompartment lungs included both healthy/uninvolved lung, as well as cellular lesion and caseum of the infected tissue. Next, the extrapolation of lung PK profiles was performed from preclinical species to humans. The approach was based on the study of Gaohua *et al*.^6^; however, the main differences are that here a middle-out interspecies extrapolation strategy is applied as opposed to the whole-body PBPK *in vitro/in vivo* extrapolation approach of Gaohua *et al.*^6^ Consequently, this PBPK approach could be used prospectively to extrapolate lung concentrations for new promising anti-TB molecules with appropriate *in vitro* and *in vivo* data (see Table S9).

Moreover, unlike empirical PK models, the developed PBPK models were able to predict the unbound concentrations in the various lung compartments where bacteria reside. Contrary to the common belief that plasma free-drug concentration is a good surrogate for tissue unbound concentration, differences for some drugs were found in our analysis. These differences concern drugs with significantly different ionization between EW and cellular lesion compartments, as well as limited exposure to the hard-to-reach caseum. These results could help multidrug regimens, leading to *in silico* clinical trials and rational model-informed drug development (MIDD). Of course, *in silico* clinical trials would carry out only part of the exploratory programme by modelling and simulation, while confirmatory trials would remain essential for the new most promising treatments.

More particularly, regarding older antibiotics, the developed PBPK approach, having a systems viewpoint across drug classes, enables the prediction and validation of unbound drug tissue concentrations over time of currently used antitubercular drugs for which lung data through biopsies are available. Such drugs are rifampicin, isoniazid, linezolid, moxifloxacin, clofazimine, pyrazinamide and kanamycin.^17^ Utilizing the literature empirical lung PK profiles of these drugs, the appropriate parameters through our PBPK models could be fitted so that the fitted lung PK profiles closely match the respective literature empirical ones. In this way, the estimated unbound drug concentrations over time of these older compounds in the various lung compartments could be used as input in PD experiments, such as a hollow-fibre system model for TB, to support much more appropriate dose selection.

On the other hand, the exposure of new promising antibiotics in the various microenvironments of the TB-infected lung should be considered for the selection of new dosing regimens. More particularly, new drug combinations should target sufficient exposure of at least one drug in different compartments where bacteria reside. Conclusively, appropriate lung PK is needed to translate *in vitro* and preclinical *in vivo* results in the clinic and this PBPK approach offers a way to do it in a stepwise, mechanistic approach.

## Supplementary Material

dkae109_Supplementary_Data

## Supplementary data

The method development in detail, Figures S1 to S14 and Tables S1 to S12 are available as Supplementary data at *JAC* Online.

## References

[1] WHO . Global Tuberculosis Report. 2023. https://iris.who.int/bitstream/handle/10665/373828/9789240083851-eng.pdf?sequence=1.

[2] Jindani A, Harrison TS, Nunn AJ et al High-dose rifapentine with moxifloxacin for pulmonary tuberculosis. N Engl J Med 2014; 371: 1599–608. 10.1056/NEJMoa1314210 25337749 PMC4233406

[3] Dartois V . The path of anti-tuberculosis drugs: from blood to lesions to mycobacterial cells. Nat Rev Microbiol 2014; 12: 159–67. 10.1038/nrmicro3200 24487820 PMC4341982

[4] Prideaux B, Via LE, Zimmerman MD et al The association between sterilizing activity and drug distribution into tuberculosis lesions. Nat Med 2015; 21: 1223–7. 10.1038/nm.3937 26343800 PMC4598290

[5] Pienaar E, Dartois V, Linderman JJ et al *In silico* evaluation and exploration of antibiotic tuberculosis treatment regimens. BMC Syst Biol 2015; 9: 79. 10.1186/s12918-015-0221-8 26578235 PMC4650854

[6] Gaohua L, Wedagedera J, Small BG et al Development of a multicompartment permeability-limited lung PBPK model and its application in predicting pulmonary pharmacokinetics of antituberculosis drugs. CPT Pharmacometrics Syst Pharmacol 2015; 4: 605–13. 10.1002/psp4.12034 26535161 PMC4625865

[7] Peters S . Physiologically-Based Pharmacokinetic (PBPK) Modeling and Simulations: Principles, Methods, and Applications in the Pharmaceutical Industry. John Wiley & Sons, 2012. 10.1038/psp.2013.29 PMC3733532 23842098

[8] Prideaux B, Dartois V, Staab D et al High-sensitivity MALDI-MRM-MS imaging of moxifloxacin distribution in tuberculosis-infected rabbit lungs and granulomatous lesions. Anal Chem 2011; 83: 2112–8. 10.1021/ac1029049 21332183 PMC3158846

[9] Irwin SM, Prideaux B, Lyon ER et al Bedaquiline and pyrazinamide treatment responses are affected by pulmonary lesion heterogeneity in *Mycobacterium tuberculosis* infected C3HeB/FeJ mice. ACS Infect Dis 2016; 2: 251–67. 10.1021/acsinfecdis.5b00127 27227164 PMC4874602

[10] Muliaditan M, Teutonico D, Ortega-Muro F et al Prediction of lung exposure to anti-tubercular drugs using plasma pharmacokinetic data: implications for dose selection. Eur J Pharm Sci 2022; 173: 106163. 10.1016/j.ejps.2022.106163 35248733

[11] Rifat D, Prideaux B, Savic RM et al Pharmacokinetics of rifapentine and rifampin in a rabbit model of tuberculosis and correlation with clinical trial data. Sci Transl Med 2018; 10: eaai7786. 10.1126/scitranslmed.aai7786 29618565 PMC5969904

[12] Zimmerman M, Lestner J, Prideaux B et al Ethambutol partitioning in tuberculous pulmonary lesions explains its clinical efficacy. Antimicrobic Agents Chemother 2017; 61: e00924-17. 10.1128/AAC.00924-17 PMC5571334 28696241

[13] Sarathy J, Blanc L, Alvarez-Cabrera N et al Fluoroquinolone efficacy against tuberculosis is driven by penetration into lesions and activity against resident bacterial populations. Antimicrob Agents Chemother 2019; 63: e02516-18. 10.1128/AAC.02516-18 30803965 PMC6496041

[14] Pienaar E, Sarathy J, Prideaux B et al Comparing efficacies of moxifloxacin, levofloxacin and gatifloxacin in tuberculosis granulomas using a multi-scale systems pharmacology approach. PLoS Comput Biol 2017; 13: e1005650. 10.1371/journal.pcbi.1005650 28817561 PMC5560534

[15] Blanc L, Daudelin IB, Podell BK et al High-resolution mapping of fluoroquinolones in TB rabbit lesions reveals specific distribution in immune cell types. Elife 2018; 7: e41115. 10.7554/eLife.41115 30427309 PMC6249001

[16] Peloquin CA, Bulpitt AE, Jaresko GS et al Pharmacokinetics of ethambutol under fasting conditions, with food, and with antacids. Antimicrob Agents Chemother 1999; 43: 568–72. 10.1128/AAC.43.3.568 10049268 PMC89161

[17] Strydom N, Gupta SV, Fox WS et al Tuberculosis drugs’ distribution and emergence of resistance in patient’s lung lesions: a mechanistic model and tool for regimen and dose optimization. PLoS Med 2019; 16: e1002773. 10.1371/journal.pmed.1002773 30939136 PMC6445413

[18] Conte JE Jr, Golden JA, Kipps J et al Effects of AIDS and gender on steady-state plasma and intrapulmonary ethambutol concentrations. Antimicrob Agents Chemother 2001; 45: 2891–6. 10.1128/AAC.45.10.2891-2896.2001 11557486 PMC90748

[19] Rodgers T, Leahy D, Rowland M. Physiologically-based pharmacokinetic modelling 1: predicting the tissue distribution of moderate-to-strong bases. J Pharm Sci 2005; 94: 1259–76. Erratum in: J Pharm Sci 2007; 96: 3151–2. 10.1002/jps.20322 15858854

[20] Rodgers T, Rowland M. Physiologically-based pharmacokinetic modelling 2: predicting the tissue distribution of acids, very weak bases, neutrals and zwitterions. J Pharm Sci 2006; 95: 1238–57. Erratum in: J Pharm Sci 2007; 96: 3153–4. 10.1002/jps.20502 16639716

[21] Rodgers T, Rowland M. Mechanistic approaches to volume of distribution predictions: understanding the processes. Pharm Res 2007; 24: 918–33. 10.1007/s11095-006-9210-3 17372687

[22] Kaya F, Ernest JP, LoMauro K et al A rabbit model to study antibiotic penetration at the site of infection for nontuberculous mycobacterial lung disease: macrolide case study. Antimicrob Agents Chemother 2022; 66: e0221221. 10.1128/aac.02212-21 35099272 PMC8923217

[23] Sarathy JP, Zuccotto F, Hsinpin H et al Prediction of drug penetration in tuberculosis lesions. ACS Infect Dis 2016; 2: 552–63. 10.1021/acsinfecdis.6b00051 27626295 PMC5028112

[24] Kempker RR, Heinrichs MT, Nikolaishvili K et al Lung tissue concentrations of pyrazinamide among patients with drug-resistant pulmonary tuberculosis. Antimicrob Agents Chemother 2017; 61: e00226-17. 10.1128/AAC.00226-17 28373198 PMC5444116

[25] Mor N, Simon B, Mezo N et al Comparison of activities of rifapentine and rifampin against *Mycobacterium tuberculosis* residing in human macrophages. Antimicrob Agents Chemother 1995; 39: 2073–7. 10.1128/AAC.39.9.2073 8540718 PMC162883

[26] Litjens CHC, Verscheijden LFM, Bolwerk C et al Prediction of moxifloxacin concentrations in tuberculosis patient populations by physiologically based pharmacokinetic modeling. J Clin Pharmacol 2022; 62: 385–96. 10.1002/jcph.1972 34554580 PMC9297990

[27] Olsson B, Bondesson E, Borgström L et al Controlled pulmonary drug delivery. In: Smyth H, Hickey A, ed. Pulmonary Drug Metabolism, Clearance, and Absorption. Springer, 2011; 21–50.

[28] Monolix . Population Parameter Estimation Using SAEM. 2023. https://monolix.lixoft.com/tasks/population-parameter-estimation-using-saem/.

[29] Reali F, Fochesato A, Kaddi C et al A minimal PBPK model to accelerate preclinical development of drugs against tuberculosis. Front Pharmacol. 2024; 14: 1272091. 10.3389/fphar.2023.1272091 38239195 PMC10794428

[30] Chen C, Ortega F, Alameda L et al Population pharmacokinetics, optimized design and sample size determination for rifampicin, isoniazid, ethambutol and pyrazinamide in the mouse. Eur J Pharm Sci 2016; 93: 319–33. 10.1016/j.ejps.2016.07.017 27473307

[31] Chen C, Wicha SG, de Knegt GJ et al Assessing pharmacodynamic interactions in mice using the multistate tuberculosis pharmacometric and general pharmacodynamic interaction models. CPT Pharmacometrics Syst Pharmacol 2017; 6: 787–97. 10.1002/psp4.12226 28657202 PMC5702905

[32] Wagner C, Pan Y, Hsu V et al Predicting the effect of cytochrome P450 inhibitors on substrate drugs: analysis of physiologically based pharmacokinetic modeling submissions to the US Food and Drug Administration. Clin Pharmacokinet 2015; 54: 117–27. 10.1007/s40262-014-0188-4 25260695

[33] Tewes F, Brillault J, Couet W et al Formulation of rifampicin-cyclodextrin complexes for lung nebulization. J. Control Release 2008; 129: 93–9. 10.1016/j.jconrel.2008.04.007 18514353

[34] Brillault J, De Castro WV, Harnois T et al P-glycoprotein-mediated transport of moxifloxacin in a Calu-3 lung epithelial cell model. Antimicrob Agents Chemother 2009; 53: 1457–62. 10.1128/AAC.01253-08 19188390 PMC2663113

[35] Barot M, Gokulgandhi MR, Pal D et al *In vitro* moxifloxacin drug interaction with chemotherapeutics: implications for retinoblastoma management. Exp Eye Res 2014; 118: 61–71. 10.1016/j.exer.2013.10.009 24157270 PMC3918950

[36] Mairinger S, Hernández-Lozano I, Filip T et al Influence of P-glycoprotein on pulmonary disposition of the model substrate [^11^C]metoclopramide assessed by PET imaging in rats. Eur J Pharm Sci 2023; 183: 106404. 10.1016/j.ejps.2023.106404 36773747

