# Correction to: Extrapolation of lung pharmacokinetics of antitubercular drugs from preclinical species to humans using PBPK modelling

**DOI:** 10.1093/jac/dkae251

**Published:** 2024-07-10

This is a correction to: Evangelos Karakitsios, Aristides Dokoumetzidis, Extrapolation of lung pharmacokinetics of antitubercular drugs from preclinical species to humans using PBPK modelling, *Journal of Antimicrobial Chemotherapy*, Volume 79, Issue 6, June 2024, Pages 1362–1371, https://doi.org/10.1093/jac/dkae109

The supplementary data has been updated to change erroneous references to ‘liver’ to ‘lung’ on lines 41, 43, 195, 196, 215, 216, 229, and 230.

