# Supplementary material for: Extrapolation of lung pharmacokinetics of antitubercular drugs from preclinical species to humans using PBPK modelling
Source: J Antimicrob Chemother. 2024 Apr 10;79(6):1362–71. doi: 10.1093/jac/dkae109 (PMC11144487; doi:10.1093/jac/dkae109)
Supplement: dkae109_Supplementary_Data [file dkae109_supplementary_data.docx]

**Supplementary Data**

The Supplementary Data is divided in seven sections, namely “Method Development”, “Physiological Parameters”, “Drug-Related Parameters”, “Appropriate Data for lung PK Extrapolation”, “Fitted Lung PK Profiles of both the Healthy/Uninvolved and the Infected Tissue utilizing Preclinical Lung PK Profiles”, “Extrapolation of Lung PK Profiles from Preclinical Species to Humans” and “Predicted Unbound Fractions”.

# Section 1: Method Development

In this section of the supplementary material, a detailed description of the developed methodology for the PBPK models of the lung in preclinical species and their extrapolation to humans is reported.

**PBPK models for the healthy/uninvolved lung**

A middle-out PBPK approach was utilized in our exercise since the focus was on the lung distribution given that the plasma PK is known for all available drugs and species. Furthermore, whole-body PBPK models were found to produce higher deviations than middle-out PBPK models in our predictions from the observed data. In this middle-out approach empirical models were used to characterize the plasma PK of the drug. A depot compartment is used when the drug is administered orally, while the central compartment describes either the blood or plasma PK of the drug. When necessary, the central compartment is connected to peripheral compartments to capture the distribution of the drug.

Additionally, the central compartment was connected to a multi-compartment lung that is divided into three compartments – the pulmonary blood (PB), the extracellular water (EW) and the intracellular water (IW). Note that there is no mass balance between the empirical PK model and the PBPK part of the model. This parameterization is in line with the widely used mechanistic model by Rodgers and Rowland. ^1-3^ Moreover, permeability limited transfer was assumed between the extracellular and intracellular water within the lung according to the multi-compartment permeability-limited PBPK model that Jamei *et al*. built in 2014 for the liver. ^4^ This implies that only unionized and unbound drugs are able to permeate the membranes of the lung cells. Furthermore, transporters are only capable of acting on unbound drugs.

Furthermore, an instantaneous equilibrium was assumed between the vascular and the extracellular compartments in our lung model in accordance with the liver permeability limited PBPK model. ^4^ This equilibrium assumes that, at any time, unbound unionized drug concentrations in these two compartments are equal. Hence, the transport of the unbound, unionized drug from the vascular space to the EW is not assumed to be a rate-limiting factor. It is noted that even though the vascular and extracellular compartments are in instantaneous equilibrium, the total concentration in these compartments can be different. ^4^

The respective differential equations for the EW and the IW are shown in equations (1) and (2):

| $\left( V_{EW}+\frac{V_{PB}}{K_{EW:B}} \right)*\left( \frac{1}{{fu}_{EW}} \right)*\frac{{dCu}_{EW}}{dt}=Q_{lung}*C_{blood}-\left( Q_{lung} \right)*\left( \frac{1}{{fu}_{EW}} \right)*\frac{{Cu}_{EW}}{K_{EW:B}}+$  $+ \mathrm{Perm}_{\mathrm{Cells}}*\mathrm{SA}_{\mathrm{Cells}}*\left( \frac{{Cu}_{IW}}{a}-\frac{{Cu}_{EW}}{W} \right)$ | (1) |
| --- | --- |

| $\left( V_{lung}*f_{IW} \right)*\left( \frac{1}{{fu}_{IW}} \right)*\frac{{dCu}_{IW}}{dt}=$ $\mathrm{Perm}_{\mathrm{Cells}}*\mathrm{SA}_{\mathrm{Cells}}*\left( \frac{{Cu}_{EW}}{W}-\frac{{Cu}_{IW}}{a} \right)$ | (2) |
| --- | --- |

where V_EW_, V_PB_ and V_lung_ are the volumes of EW, PB and lung respectively, K_EW:B_ is the quotient of total EW concentration and the total blood concentration in the tissue vascular space. ^4^ Cu_EW_ and Cu_IW_ represent the unbound EW and IW concentrations, respectively, while fu_EW_ and fu_IW_ are the extracellular and intracellular unbound fractions respectively. Also, Q_lung_ is the blood flow rate of the lung and C_blood_ is the blood concentration of the anti-TB drug. When plasma concentrations were available instead of blood concentrations, C_blood_ was replaced by the plasma concentrations multiplied by blood to plasma ratio.

Moreover, Perm_Cells_ is the drug’s permeability between the EW and IW and SA_Cells_ is the surface area of the lung cells. Additionally, the terms W and *a* account for drug ionization based on compound charge type using the Henderson-Hasselbalch equation.^2-3^ For example, for a monoprotic base: W is equal to 1+10^pKa−pHEW^ and *a* is equal to 10^pKa−pHIW^ where pKa is the drug acid-ionisation constant and pH_EW_ and pH_IW_ are the EW and IW pH values, respectively. Finally, f_IW_ represents the intracellular volume fraction.

It is noted that for the differential equation of the EW, an effective organ volume rather than the actual volume is used, according to Berezhkovskiy. This can be really important for organs with large perfusion volume, such as the lung tissue, as well as for drugs with small tissue to plasma partition coefficient and high blood to plasma concentration ratio. As shown in equation (1), the effective organ EW volume is a combination of the volumes of EW and PB ($V_{EW-effective}=V_{EW}+\frac{V_{PB}}{K_{EW:B}}$). The ‘‘adjusted’’ part of perfusion volume in equation (1), given as $\frac{V_{PB}}{K_{EW:B}}$, is used to account for the partitioning between PB and EW inside the organ.^5^

Considering the tissue binding of drugs within the lung, the following assumptions were made that originate from the Rodgers and Rowland method. ^1-3^ First, in plasma and in EW the drug binds reversibly to proteins (mostly albumin). Also, in IW the drug partitions between lipids (neutral lipids, neutral phospholipids and acidic phospholipids) and water. Moreover, only the unbound, ionized fraction of the drug, in IW, can bind to the acidic phospholipids. Additionally, for moderate-to-strong bases (molecules with at least one basic pK_a_ ≥ 7), binding to acidic phospholipids is assumed to dominate the distribution. On the other hand, for all other compounds (molecules with no basic pK_a_ ≥ 7) binding to extracellular proteins is assumed to predominate.

Our general strategy, as stated previously, was to initially build permeability-limited lung PBPK models in small preclinical species. Then, we attempted to find the key distribution parameters for each drug and try to scale the physiological parameters and extrapolate the lung PK to humans. Regarding the drugs for which healthy/uninvolved lung PK profiles were available in the literature in at least two species, rifampicin, ethambutol and moxifloxacin are moderate-to-strong bases (pK_a_ ≥ 7) and hence will bind within the intra-cellular space. On the contrary, pyrazinamide and isoniazid are weak bases and consequently will bind within the extracellular space.

Finally, in the case of moderate-to-strong bases fu_EW_ is assumed to be 1 based on Rodgers and Rowland theory, while the cellular permeability (Perm_cells_) as well as fu_IW_ are fitted using lung PK profiles. On the other hand, in the case of weak bases, fu_IW_ is approximately 1, since based on Rodgers and Rowland theory binding to extracellular proteins is assumed to predominate and the affinity constant for the acidic phospholipids is assumed to be 0. Furthermore, for weak bases, fu_EW_ in humans, is estimated from the fraction unbound in plasma, as described subsequently, while the cellular permeability (Perm_cells_) is fitted again using lung PK profiles. It is noted that, in each case of fitting, constant, proportional and combined error models were tried. In addition, generally, an instant equilibrium is assumed between the PB and the EW. However, in the case of moxifloxacin, this assumption was not found to be correct and hence permeability limited transfer was introduced between these two compartments. Therefore, surface areas of capillaries (SA_capillaries_) and capillary permeability values (Perm_capillaries_) were also included in the model of moxifloxacin. The specific parameters for each species and each drug are provided in the following sections (Physiological Parameters and Drug-Related Parameters).

Considering the extrapolation step, all the permeability values, either cellular (Perm_cells_) or capillary (Perm_capillaries_) were kept constant across species taking into account the different surface areas of the species, either cellular or capillary respectively. It is noted that an instant equilibrium between the PB and EW of the lung compartment was assumed for rifampicin, ethambutol, pyrazinamide and isoniazid. For these compounds, only cellular permeability and surface area values were used.

However, such an instant equilibrium could not be used for moxifloxacin since moxifloxacin is a substrate of p-glycoprotein (P-gp).^6^ P-gp is an efflux transporter on the apical membrane of the pulmonary epithelial and vascular endothelial cells and therefore a time-independent distribution of moxifloxacin between PB and EW cannot be considered valid.^7^ Consequently, for moxifloxacin two separate compartments were used for PB and EW. The respective differential equations for moxifloxacin are shown in equations (3) and (4):

| $\left( V_{PB} \right)\frac{{dC}_{PB}}{dt}=Q_{lung}*C_{blood}-\mathrm{Perm}_{\mathrm{Capillaries}}*{SA}_{\mathrm{Capillaries}}*$  $*\left( \frac{{Cu}_{EW}}{W}-{fu}_{pls}*{fni}_{plasma}*\left( \frac{C_{PB}}{B:P} \right) \right)$ | (3) |
| --- | --- |

| $\left( V_{EW} \right)*\left( \frac{1}{{fu}_{EW}} \right)*\frac{{dCu}_{EW}}{dt}={Perm}_{Capillaries}*{SA}_{Capillaries}*\left( \frac{{Cu}_{EW}}{W}-{fu}_{pls}*{*fni}_{plasma}*\left( \frac{C_{PB}}{B:P} \right) \right)+\mathrm{Perm}_{\mathrm{Cells}}*\mathrm{SA}_{\mathrm{Cells}}*\left( \frac{{Cu}_{IW}}{a}-\frac{{Cu}_{EW}}{W} \right)$ | (4) |
| --- | --- |

where V_PB_ is the volume of PB, C_PB_ is the concentration in PB, Perm_Capillaries_ is the drug’s permeability between the PB and EW and SA_Capillaries_ is the surface area of the capillaries. Additionally, fu_pls_ is the fraction unbound in plasma, fni_pls_ is the non-ionized fraction of drug in plasma and B:P is the drug’s blood to plasma ratio.

Furthermore, regarding the case of moderate-to-strong bases (rifampicin, ethambutol and moxifloxacin), as stated earlier, fu_IW_ in lung was fitted using lung PK profiles in a small preclinical species, either mice or rabbits. Utilizing this fitted fu_IW_ as well as the mechanistic equation that has been provided for fu_IW_ by Jamei *et al*., 2014, the affinity constant for the acidic phospholipids in lung (Ka_AP_) was obtained, as shown in equations (5) and (6).^3^ This affinity constant was assumed to have the same value across species and was used to calculate the fu_IW_ in humans. It is noted that different physiological values were used for each species and different drug-related parameters were used for each anti-TB, as mentioned previously.

| ${fu}_{IW}=\frac{f_{IW}}{f_{IW}+\frac{P*f_{NL}+\left( 0.3*P+0.7 \right)*f_{NP}+{Ka}_{AP}*\left[ AP \right]_{T}*a}{1+a}}$ | (5) |
| --- | --- |

| ${Ka}_{AP}=\frac{f_{IW}*\left( \frac{1+a}{{fu}_{IW}}-1 \right)-\left[ P*f_{NL}+\left( 0.3*P+0.7 \right)*f_{NP} \right]}{\left[ AP \right]_{T}*a}$ | (6) |
| --- | --- |

where P is the n-octanol:water partition coefficient, f_NL_ is the relative volume (%) of neutral lipids in lung, f_NP_ is the relative volume (%) of neutral phospholipids in lung and [AP]_T_ is the tissue concentration of acidic phospholipids in lung (mg/g).

Finally, regarding the case of weak bases (pyrazinamide and isoniazid), fraction unbound in the EW of the lung in humans was predicted based on the drug’s fraction unbound in plasma. According to Johnson and Smith, within the therapeutic range of the drugs’ concentrations, isoniazid and pyrazinamide will bind to human serum albumin.^8^ Two main assumptions were made for this prediction of fu_EW_. First, albumin was assumed to be the dominant binding protein in tissues for these weak bases and second both ionized and unionized molecules were assumed capable of interacting with albumin.^4^

Initially, through the equation that provides the unbound fraction in plasma fu_pls_ (equation (7)), the affinity constant for human serum albumin (HSA) was obtained (Ka^HSA^), as shown in equation (8). ^9^

| ${fu}_{pls}=\frac{1}{1+\frac{[HSA]}{K_{D}^{HSA}}}$ | (7) |
| --- | --- |
| $Ka^{HSA}=\frac{1}{K_{D}^{HSA}}=\frac{\frac{1}{{fu}_{plasma}}-1}{[HSA]}$ | (8) |

where $K_{D}^{HSA}$ is the dissociation constant of binding to albumin and [HSA] is the concentration of albumin in human serum. Eventually, fraction unbound in the lung’s EW in humans was predicted based on the equation provided by Jamei *et al*., 2014 (equation (9)):

| $\mathrm{fu}_{\mathrm{EW}}=\frac{1}{1+\frac{Ka^{HSA}*{[HSA]}_{\mathrm{lung}}}{f_{\mathrm{EW}}}}$ | (9) |
| --- | --- |

where [HSA]_lung_ is the concentration of HSA in lung and f_EW_ represents the extracellular volume fraction of the lung in humans.

**PBPK models for the infected lung**

The respective differential equations for the cellular lesion and the caseum are depicted in equations (10) and (11):

| $\frac{{dC}_{Lesion}}{dt}=\mathrm{rate}_{\mathrm{Lesion}}*\left( \frac{{Cu}_{EW}}{W}-{fni}_{Lesion}*{fu}_{Lesion}*C_{Lesion} \right)$ | (10) |
| --- | --- |

| $\frac{{dC}_{Caseum}}{dt}=$ $\mathrm{rate}_{\mathrm{Caseum}}*\left( {PC}_{Caseum-Lesion}*C_{Lesion}- C_{Caseum} \right)$ | (11) |
| --- | --- |

Where C_Lesion_ and C_Caseum_ represent the concentrations of cellular lesion and caseum respectively. Also, fu_Lesion_ and fu_Caseum_ are the unbound fractions of cellular lesion and caseum respectively, while fni_Lesion_ is the nonionized fraction of drug in cellular lesions. Additionally, rate_Lesion_ and rate_Caseum_ are the rates of the drug’s distribution into the cellular lesion and the caseum respectively. Finally, PC_Caseum-Lesion_ is the partition coefficient or ratio of drug’s concentration in caseum to the drug’s concentration in cellular lesion at steady state.

Note that, regarding equation (10), there is no mass balance between the cellular lesion and the caseum in our model. All the caseum concentrations in the model refer to cavity caseum and not closed nodule caseum. The volume of cavity caseum, unlike the closed nodule caseum, is significantly lower compared to the volume of the adjacent cellular lesion.^35,38^ If mass balance was applied in equation (10), then assuming rate_Lesion_ = Perm_Lesion_*SA_Lesion_/V_Lesion_ and rate_Caseum_ = Perm_Caseum_*SA_Caseum_/V_Caseum_, the following equation would be obtained:

| V_Lesion_∗dC_Lesion_/dt$=$Perm_Lesion_*SA_Lesion_*((Cu_EW_/W-fni_Lesion_*fu_Lesion_*C_Lesion_)-  -Perm_Caseum_*SA_Caseum_*(PC_Caseum-Lesion_*C_Lesion_-C_Caseum_)) | (12) |
| --- | --- |

Hence:

| dC_Lesion_/dt$=$rate_Lesion_*((Cu_EW_/W-fni_Lesion_*fu_Lesion_*C_Lesion_)-  -(rate_Caseum_*V_Caseum_/V_Lesion_)*(PC_Caseum-Lesion_*C_Lesion_-C_Caseum_)) | (13) |
| --- | --- |

Finally, assuming that V_Caseum_ is significantly lower compared to V_Lesion_, equation (10) of the paper can be obtained which is a reasonable approximation given the fact that actual volume sizes are not available. Furthermore, concerning equation (11), assuming rate_Caseum_ = Perm_Caseum_*SA_Caseum_/V_Caseum_, as stated previously, mass balance is obtained.

Regarding the extrapolation step for the infected tissue, using the PK profiles in preclinical species the two rates (rate_Lesion_ and rate_Caseum_), the product (fni_Lesion_*fu_Lesion_) as well as the partition coefficient PC_Caseum-Lesion_ were optimized. Again, constant, proportional and combined error models were tried for the optimizations. The two rates and the partition coefficient were kept constant across species. Moreover, for humans a mean pH value equal to 5.84 was used in cellular lesions to calculate the fni_Lesion_.^10^ Additionally, the *in vitro* literature Intracellular to Extracellular ratios (I/E) for macrophages was utilized to calculate the unbound fraction in the cellular lesions for moxifloxacin, rifampicin and pyrazinamide, as shown in Equation (14):^11-13^

| $\mathrm{fu}_{\mathrm{Lesion}}=\frac{\mathrm{fu}_{\mathrm{EW}}*\mathrm{fni}_{\mathrm{EW}}}{\mathrm{fni}_{\mathrm{Lesion}}*(I/E)}$ | (14) |
| --- | --- |

Moreover, concerning the caseum PK profiles, *in vitro* surrogate fraction unbound values in caseum were utilized from the literature, produced from human THP1 macrophages, to predict the unbound caseum concentrations over time in humans.^14^

All model assumptions are summarized in the following Table S1, with a detailed description and possible implications.

Table S1: Model assumptions

| **Model Assumtpion** | **Description** | **Implications** |
| --- | --- | --- |
| Predominant binding of strong-moderate bases within the intracellular space of lung. | Based on Rodgers and Rowland, strong-moderate bases bind predominantly to the acidic phospholipids of intracellular space. It is noted that only unbound, ionized fraction of the drug can bind to the acidic phospholipids in IW. | This may not apply to drugs that are strong-moderate bases but have strong affinity for albumin, such as rifampicin. Nevertheless, total cellular lesion and caseum PK profiles or unbound concentrations over time (in EW, cellular lesion and caseum) of rifampicin will be unaffected. It is noted that the PBPK models are parameterized in such a way that at any moment the unbound concentrations in EW and plasma are equal (given the same pH in these two compartments). Hence, any change in fu_EW_ will impact total concentrations in EW (C_EW_), but always the product of fu_EW_ times C_EW_ will be equal to unbound plasma concentrations. These concentrations are then connected, through differential equations, to further compartments in the model. |
| Predominant binding of weak bases within the interstitial fluid of lung. | Based on Rodgers and Rowland, weak bases bind predominantly to the albumin of extracellular space. | Isoniazid and pyrazinamide are weak bases that are almost neutral within intracellular space. Hence, their binding to acidic phospholipids will be minimal. |
| Instant equilibrium between PB and EW. | Unbound, unionized drug concentrations in these two compartments are equal at any time | This assumption has been utilized for other tissues, such as the liver.^4^ However, in cases where the drug is a substrate of a transporter lying on the surface of pulmonary capillaries, this assumption is not valid (e.g. moxiflxoacin).^16^ |
| Permeability limited transfer between EW and healthy/uninvolved pulmonary cells (IW) as well as macrophages (cellular lesion) | Only unbound, unionised drugs can permeate the cellular membrane of pulmonary cells and macrophages | Basic assumption for permeation of cellular membranes by drugs, that has been applied also to other tissues. .^4^ |
| Passive diffusion of drugs from the cellular lesion to the caseum | Drugs passively diffuse from the outer cellular rim towards the necrotic center of the caseum. Also, the binding of drugs to macromolecules at the outer caseum prevents their further diffusion towards the inner caseous center.^14,15^ | Due to lack of relevant data (laser capture microdissection of different regions of the caseum), anomalous diffusion was not considered in the model. Caseum was considered as a homogeneous medium. This could have a major impact in drugs that diffuse very slowly into the caseum, like bedaquiline. |
| Healthy/uninvolved lung included pulmonary capillaries | Predicted healthy/uninvolved lung homogenate concentrations included pulmonary blood to model the respective output lung concentrations | The volume of pulmonary blood is high in all species, and this could impact PBPK predictions concerning healthy/uninvolved lung. |
| Cellular lesion data do not contain any EW during the sample acquisition | Cellular lesions are in direct contact with EW and, hence, the samples could contain some levels of EW | The volume of EW within lesions is not anticipated to be significant. Extracellular collagen matrix is increasingly depleted in the cavity wall moving toward the necrotic center of caseous lesions.^17^ |
| There is no mass balance between the empirical PK model and the lung model. | In our middle-out PBPK model, a pulmonary blood flow rate is used to transfer drugs from the central compartment (plasma/blood) of the empirical PK model to the pulmonary blood.  Hepatic metabolism and biliary/renal clearance are accounted for in the empirical plasma PK model. | Metabolism in lung tissue does not contribute significantly to the overall elimination of the antibiotics used in the current analysis. In case a drug is metabolised in the lung, the differential equations of the middle-out PBPK model could be rearranged to describe such phenomena. Additionally, since lung volume is low compared to the whole volume of organisms, the inclusion of lung in the empirical plasma/blood PK model is not anticipated to have a significant impact on volume of distribution at steady state. |

# Section 2: Physiological Parameters

Table S2. Physiological parameters of mice

| **Species: Mouse** | | |
| --- | --- | --- |
| **Parameter** | **Value** | **Reference** |
| Body Weight (kg) | 0.025 | 18 |
| Volume of Pulmonary Blood (mL^a^) | 0.03135 | 18,19,20 |
| Blood Flow rate (mL/min) | 14.01 | 18 |
| Extracellular Volume of Lung Tissue (mL)^b^ | 0.019155 | 18 |
| Intracellular Volume of Lung Tissue (mL)^c^ | 0.099495 | 18 |
| Surface Area of lung cells (m^2^) | 0.05 | 21 |
| Surface Area of capillaries in lung (m^2^) | 0.04 | 21 |
| pH_plasma_ | 7.391 | 22 |
| pH_EW-Lung_ | 7.4 | 18 |
| pH_IW-Lung_ | 7.36 | 18 |
| Relative volume of intracellular lung tissue (%) | 44.69 | 18 |
| Relative volume of neutral lipids in lung (%) | 1.9 | 18 |
| Relative volume of neutral phospholipids in lung (%) | 1.35 | 18 |
| Tissue Concentration  of Acidic Phospholipids in lung (mg/g) | 2.6 | 18 |

^a^The volume of pulmonary blood is proportional to body weight.^20^

^b^The Extracellular volume is equal to the difference between the fraction of extracellular space times the lung volume minus the volume of pulmonary blood

^c^The intracellular volume is equal to the lung volume minus the sum of pulmonary blood and extracellular volume

Table S3. Physiological parameters of rabbits

| **Species: Rabbit** | | |
| --- | --- | --- |
| **Parameter** | **Value** | **Reference** |
| Body Weight (kg) | 2.5 | 23 |
| Volume of Pulmonary Blood ( mL^a^) | 3.135 | 19,20,23 |
| Blood Flow rate ( mL/sec) | 5.5211 | 23 |
| Extracellular Volume of Lung Tissue  (mL^b^) | 2.577 | 23 |
| Intracellular Volume of Lung Tissue  ( mL^c^) | 11.288 | 23 |
| Surface Area of lung cells (m^2^) | 3.36 | 21 |
| Surface Area of capillaries in lung  ( m^2^) | 3.10 | 21 |
| pH_plasma_ | 7.389 | 23 |
| pH_EW-Lung_ | 7.4 | 23 |
| pH_IW-Lung_ | 7.14 | 24 |
| Relative volume of intracellular lung tissue (%) | 47.5 | 23 |
| Relative volume of neutral lipids in lung (%) | 0.3 | 23 |
| Relative volume of neutral phospholipids in lung (%) | 0.8 | 23 |
| Tissue Concentration  of Acidic Phospholipids in lung (mg/g) | 2.06 | 26 |

^a^The volume of pulmonary blood is proportional to body weight. ^20^

^b^The Extracellular volume is equal to the difference between the fraction of extracellular space times the lung volume minus the volume of pulmonary blood

^c^The intracellular volume is equal to the lung volume minus the sum of pulmonary blood and extracellular volume

Table S4. Physiological parameters of humans

| **Species: Human** | | |
| --- | --- | --- |
| **Parameter** | **Value** | **Reference** |
| Body Weight (kg) | 80.7 | 19 |
| Volume of Pulmonary Blood (mL) | 101.195 | 19 |
| Blood Flow rate ( L/hr) | 356 | 19 |
| Extracellular Volume of Lung Tissue (mL^a^) | 89.161 | 19 |
| Intracellular Volume of Lung Tissue (mL^b^) | 356.644 | 19 |
| Surface Area of lung cells (m^2^) | 96 | 21 |
| Surface Area of capillaries in lung  ( m^2^) | 72.3 | 21 |
| pH_plasma_ | 7.4 | 18 |
| pH_EW-Lung_ | 7.4 | 18 |
| pH_IW-Lung_ | 6.69 | 25 |
| Relative volume of intracellular lung tissue (%) | 46.3 | 18 |
| Relative volume of neutral lipids in lung (%) | 0.3 | 18 |
| Relative volume of neutral phospholipids in lung (%) | 0.9 | 18 |
| Tissue Concentration  of Acidic Phospholipids in lung (mg/g) | 0.5 | 18 |
| Relative volume of extracellular lung tissue (%) | 34.8 | 18 |
| Reference concentration for human serum albumin (g/L) in plasma | 45 | 18 |
| Lung-to-plasma  Albumin ratio | 0.212 | 18 |

^a^The Extracellular volume is equal to the difference between the fraction of extracellular space times the lung volume minus the volume of pulmonary blood

^b^The intracellular volume is equal to the lung volume minus the sum of pulmonary blood and extracellular volume

# Section 3: Drug-Related Parameters

Table S5. Drug-related Parameters of Rifampicin (RIF), Ethambutol (ETH), Moxifloxacin (MXF), Isoniazid (INH) and Pyrazinamide (PZA) in all species

| **Parameter** | **Value** | **Reference** |
| --- | --- | --- |
| Molecular Weight | RIF: 822.9 g/mol  ETH: 204.31 g/mol  MXF: 437.896 g/mol  INH: 137.14 g/mol  PZA: 123.11 g/mol | Litjens et al., 2022^6^,  PubChem ^27,32,34,36,32^ |
| pKa value | RIF: pKa_1_=1.7  pKa_2_=7.9 (ampholyte)  ETH: pKa_1_=9.55 (base)  pKa_2_=6.5 (base)  MXF: pKa_1_=6.25  pKa_2_=9.29 (ampholyte)  INH: pKa = 3.27 (base)  PZA: pKa = 3.53 (base) | Litjens et al., 2022^6^  Humphries et al., 2021^28^  PubChem ^34^ |
| Calculated logP value (lipophilicity) | RIF: 4.01  ETH: 0.059  MXF: 0.832  INH: -0.668  PZA: -0.206 | Litjens et al., 2022^6^  Humphries et al., 2021^28^  PubChem ^34^ |

Table S6. Drug-related Parameters of Rifampicin (RIF), Ethambutol (ETH), Moxifloxacin (MXF), Isoniazid (INH) and Pyrazinamide (PZA) in mice

| **Parameter** | **Value** | **Reference/Comments** |
| --- | --- | --- |
| Blood to plasma ratio | RIF: 0.58  ETH: 1.08  INH: 0.84  PZA: 0.79 | GSK data |
| fu_plasma_ | RIF: 0.029  ETH: 0.826  INH: 0.396  PZA: 0.591 | GSK data |
| fni_PB_^a^ | RIF: 4.82E-7  ETH: 6.10E-3  INH: 0.99992  PZA: 1 | Rodgers and Rowland, 2007^3^ |
| fni_EW_^a^ | RIF: 4.79E-7  ETH: 6.24E-3  INH: 0.99993  PZA: 1 | Rodgers and Rowland, 2007^3^ |
| fni_IW_^a^ | RIF: 4.90E-7  ETH: 5.64E-3  INH: 0.99992  PZA: 1 | Rodgers and Rowland, 2007^3^ |
| fu_EW_ | RIF: 1  ETH: 1  INH: 0.17  PZA: 0.25 | For RIF and ETH fu_EW_ was assumed 1, while for INH and PZA it was optimized based on *in vivo* lung PK data.^4,29^ |
| fu_IW_ | RIF: 0.0084  ETH: 0.056  INH: 0.97  PZA: 0.95 | For RIF and ETH fu_IW_ was optimized based on *in vivo* lung PK profiles, while for INH and PZA it was predicted assuming that the affinity constant for the acidic phospholipids is 0. ^4,29^ |
| Permeability of  lung cells (dm/h) | RIF: 144942.33  ETH: 0.034  INH: 0.000037  PZA: 0.000021 | Optimized based on *in vivo* lung PK profiles. ^29^ |

^a^ The fraction nonionized (fni) in pulmonary blood (PB), extracellular water (EW) or intracellular water (IW) was calculated based on the pKa value of the drug and the physiological pH value in each compartment

Table S7. Drug-related Parameters of Rifampicin (RIF), Ethambutol (ETH) and Moxifloxacin (MXF) in rabbits

| **Parameter** | **Value** | **Reference/Comments** |
| --- | --- | --- |
| Blood to plasma ratio | RIF: 0.66178  ETH: 0.92872  MXF: 0.890308 | This ratio was calculated assuming partition to the blood cells is only dependent on fu_plasma_ and hematocrit^b^. ^30^ |
| fu_plasma_ | RIF: 0.0605  ETH: 0.802  MXF: 0.695 | Sarathy et al., 2016^14^ |
| fni_vascular_^a^ | RIF: 4.82E-7  ETH: 6.07E-3  MXF: 8.40E-4 | Rodgers and Rowland, 2007^3^ |
| fni_EW_^a^ | RIF: 4.79E-7  ETH: 6.24E-3  MXF: 8.41E-4 | Rodgers and Rowland, 2007^3^ |
| fni_IW_^a^ | RIF: 5.38E-7  ETH: 3.15E-3  MXF: 8.02E-4 | Rodgers and Rowland, 2007^3^ |
| fu_EW_ | RIF: 1  ETH: 1  MXF: 1 | Jamei et al, 2014^4^ |
| fu_IW_ | RIF: 0.015  ETH: 0.08  MXF: 0.056 | fu_IW_ was optimized based on *in vivo* lung PK profiles.^11,37-39^ |
| Permeability of  lung cells (dm/h) | RIF: 3216.69  ETH: 0.023  MXF: 0.99 | This permeability was optimized based on *in vivo* lung PK profiles. ^11,37-39^ |
| Permeability of  lung capillaries (dm/h) | MXF: 0.6 | Optimized based on *in vivo* lung PK profiles^11,37^ |

^a^ The fraction nonionized (fni) in pulmonary blood (PB), extracellular water (EW) or intracellular water (IW) was calculated based on the pKa value of the drug and the physiological pH value in each compartment

^b^ The hematocit value in rabbits was derived by Davies and Moris, 1993

Table S8. Drug-related Parameters of Rifampicin (RIF), Ethambutol (ETH), Moxifloxacin (MXF), Isoniazid (INH) and Pyrazinamide (PZA) in humans

| **Parameter** | **Value** | **Reference/Comments** |
| --- | --- | --- |
| Blood to plasma ratio | RIF: 0.59  MXF: 1.045  INH: 0.89  PZA: 1.12  ETH: 1.04 | Edginton et al., 2009^33^,  GSK data |
| fu_plasma_ | RIF: 0.078  MXF: 0.7168  INH: 0.516  PZA: 0.719  ETH: 0.853 | GSK data,  PubChem^32^ |
| fni_vascular_^a^ | RIF: 4.79E-7  MXF: 8.41E-4  INH: 0.99993  PZA: 1  ETH: 6.24E-3 | Rodgers and Rowland, 2007^3^ |
| fni_EW_^a^ | RIF: 4.79E-7  MXF: 8.41E-4  INH: 0.99993  PZA: 1  ETH: 6.24E-3 | Rodgers and Rowland, 2007^3^ |
| fni_IW_^a^ | RIF: 5.94E-7  MXF: 6.67E-4  INH: 0.99962  PZA: 1  ETH: 8.38E-4 | Rodgers and Rowland, 2007^3^ |
| fu_EW_ | RIF: 1  MXF: 1  ETH: 1  INH: 0.64  PZA: 0.81 | For RIF, ETH and MXF fu_EW_ was assumed 1, while for INH and PZA it was predicted based on the equation of Jamei *et al*., 2014 and each drug’s fu_plasma_. ^4^ |
| fu_IW_ | RIF: 0.044 (from mice)  0.058 (from rabbits)  MXF: 0.191  ETH: 0.24 (from mice)  0.25 (from rabbits)  INH: 0.984  PZA: 0.98 | For RIF, MXF and ETH fu_IW_ was predicted considering that the affinity constant for the acidic phospholipids is the same across species.^4^  For INH and PZA, it was predicted assuming that the affinity constant for the acidic phospholipids is 0. ^4^ |
| Permeability of  lung cells (dm/h) | RIF: 144942.33 (from mice)  3216.69 (from rabbits)  MXF: 0.99  ETH: 0.034 (from mice)  0.023 (from rabbits)  INH: 0.000037  PZA: 0.000021 | This permeability was kept constant across species |
| Permeability of  lung capillaries (dm/h) | MXF: 0.604 | This permeability was kept constant across species |

^a^ The fraction nonionized (fni) in pulmonary blood (PB), extracellular water (EW) or intracellular water (IW) was calculated based on the pKa value of the drug and the physiological pH value in each compartment

# Section 4: Appropriate data for lung PK extrapolation

Table S9. Data required for this work divided in three categories: drug-related parameters, *in vitro* data and *in vivo* data. The use of each data (either estimation/model development step for preclinical species or extrapolation step for humans) is also presented.

| **Drug-Related Parameters** | **Use** |
| --- | --- |
| Molecular Weight | Estimation/  Model development |
| pKa values | Estimation/  Model development |
| Calculated log P value | Estimation/  Model development |
| ***In Vitro* Data** |  |
| Apparent permeability data in Calu-3 cells | Extrapolation |
| Plasma protein binding  (for each species - preclinical and humans) | Estimation/  Model development AND  Extrapolation |
| Blood to Plasma ratio  (for each species - preclinical and humans) | Estimation/  Model development AND  Extrapolation |
| Uptake in primary human macrophages for both standard differentiated macrophages and foamy macrophages | Extrapolation |
| Free caseum fraction from a human surrogate matrix from lipid loaded THP-1 macrophages | Extrapolation |
| ***In Vivo* data** |  |
| In an appropriate preclinical species (Kramnik mice or rabbits): A well-defined plasma PK profile and a less defined, but targeted lesion PK profile | Estimation/  Model development |
| Laser-capture microdissection to obtain spatial quantitation in necrotic lesions (cellular rim, outer and inner caseum) | Estimation/  Model development |
| Volumes, Surfaces Area and pH values for each lesion compartment in the above preclinical species | Estimation/  Model development |
| In humans: a well-defined plasma PK profile by conducting a phase I clinical trial | Extrapolation |

# Section 5: Fitted Lung PK Profiles, of both the Healthy/Uninvolved and the Infected Tissue, utilizing Preclinical Lung PK Profiles.

In this section, a detailed description of the developed methodology for the PBPK models of the lung in preclinical species and their extrapolation to humans is reported per compound studied. It is noted that in cases where combined fitting was performed, observed versus predicted figures are presented, along with 90% prediction intervals, to improve readability. In all other cases, the fitted profiles along with the observed data and the respective 90% prediction intervals (shown as shaded areas) are depicted.

**Rifampicin**

Concerning the extrapolation of human PK of rifampicin from rabbit data, as also stated in the main text, the empirical plasma PK in rabbits after an intravenous (IV) infusion (10 mg/kg) that has been developed by Rifat *et al*. was utilized in our model. ^38^It is noted that the observed data that were used for the fitting of the cellular lesion PK were both the cellular and cavity wall lung data. These data were grouped together to increase the number of data points (five data points in total after single and multiple dosing). Note that, since fitting was performed simultaneously for all tissue PK profiles, predicted caseum PK depends on cellular lesion PK, which is further dependent on lung PK. Hence, some predicted caseum concentrations are far off the unity line in the following observed versus predicted tissue concentrations figure.

Figure S1: Observed versus predicted concentrations of rifampicin in rabbits in lung (blue dots), cellular lesion (green dots) and caseum (brown dots). The black solid line represents the unity line, while the black dashed lines refer to the 90% prediction interval.

Furthermore, regarding extrapolation of rifampicin from mice, which is shown in Figure S2, initially, an empirical model from the literature was utilized to describe the blood PK of rifampicin in mice after an oral administration of 10 mg/kg, which is the human equivalent dose in mice.^40^

Figure S2: Diagram of fitted lung PK profile of rifampicin in mice utilizing an empirical plasma PK model as well as the mean lung concentrations of rifampicin after a single oral dose of 10 mg/kg in mice.^29, 40^ The blue line represents the fitted healthy/uninvolved lung PK profile utilizing the mean lung concentrations of mice at different time points. The blue dots in the Figure are the observed lung concentrations.

**Moxifloxacin**

Regarding the extrapolation of human PK of moxifloxacin, empirical plasma PK of the drug in rabbits that has been developed by Pienaar *et al*. was utilized in our model.^11^ It is noted that, the cavity wall data were grouped together with the cellular lesion data, to increase the limited number of concentrations over time (4 data points in total). Moreover, to enrich the limited dataset we digitized and utilized the data that has been obtained from laser capture microdissection coupled with standard mass spectrometry in rabbit lesions after an oral dose of 100 mg/kg, namely the normal lung data as well as the cellular areas and the caseum of caseous granulomas.^37, 41^ It is noted that to validate the extrapolated profiles in the infected tissue, the cavity wall and the cavity caseum profiles in humans were utilized since these profiles better matched the cellular areas and the caseum of caseous granulomas respectively in rabbits. The respective observed versus predicted tissue concentrations are shown in the following Figure S3.

Figure S3: Observed versus predicted concentrations of moxifloxacin in rabbits in lung (blue dots), cellular lesion (green dots) and caseum (brown dots). The black solid line represents the unity line, while the dashed lines refer to the 90% prediction interval.

**Ethambutol**

As far as ethambutol is concerned, the lung PK extrapolation was performed from both rabbits and mice to humans. The only observed lung concentrations that were available in the literature were the lung concentrations in alveolar cells from healthy individuals after a single oral administration of 15 mg/kg. ^42^Hence, to validate the PBPK model we simulated the lung concentration only in the intracellular compartment of the lung and compared it to the mean alveolar cell concentrations of healthy individuals. It is noted that, concerning the extrapolation from mice to humans, the oral dose of 100 mg/kg that was administered in mice was utilized since this is the human equivalent dose in mice for ethambutol. ^43^

Regarding the extrapolation of human PK of ethambutol from rabbit data, an empirical plasma PK model after an oral administration of 100 mg/kg in rabbits was developed in Monolix 2021R1 utilizing the literature data by Zimmerman *et al*. ^39^The final model was a two-compartment model with first order absorption and a proportional error model. The final parameters of the model can be found in Table S10, while the respective visual predictive check is shown in Figure S4.

Table S10: Plasma PK parameters of ethambutol in rabbits

| PARAMETER | VALUE | sTANDARD  ERROR | RELATIVE STANDARD  ERROR (%) | |
| --- | --- | --- | --- | --- |
| Fixed Effects | | | | |
| Ka (1/h) | 0.76 | 0.29 | | 38.4 |
| Cl (mL/h) | 23503.5 | 2738.83 | | 11.7 |
| V1 (mL) | 18280.47 | 10239.59 | | 56.0 |
| Q (mL/h) | 7643.31 | 3991.45 | | 52.2 |
| V2 (mL) | 66960.95 | 8005.2 | | 12.0 |
| Standard Deviation of the Random Effects | | | | |
| omega_Cl | 0.34 | 0.09 | | 26.1 |
| omega_V1 | 0.15 | 0.12 | | 81.7 |
| omega_Q | 1.23 | 0.55 | | 44.8 |
| Error Model Parameters | | | | |
| b | 0.18 | 0.029 | | 16.4 |

Abbreviations: Ka, absorption rate constant; Cl, clearance of elimination; Q, inter-compartmental clearance; V1, volume of distribution of first compartment; V2, volume of distribution of second compartment; omega, standard deviation of the respective random effect referring to the Inter-individual Variability of rabbits; b, proportional error term.

Figure S4: Visual predictive check of the population plasma PK model of ethambutol in rabbits, utilizing the plasma PK data by Zimmerman *et al*. after single and multiple oral doses of 100 mg/kg. ^39^ The figure shows the empirical 10^th^,50^th^ and 90^th^ percentiles (blue solid lines) and the five and ninety-five percentiles (shaded areas); dots represent the experimental data at each time

The fitting of ethambutol’s lung PK in rabbits is shown in Figure S5 (observed versus predicted lung tissue concentrations). Furthermore, regarding the fitting of ethambutol’s lung PK in mice, following the same approach, first the blood PK of the drug after an oral administration of 100 mg/kg in mice was described using an empirical model from the literature. ^40^ The final fitting of the drug’s lung PK in mice is depicted in Figure S6.

Figure S5: Observed versus predicted lung (blue dots) concentrations of ethambutol in rabbits. The black solid line represents the unity line, while the black dashed lines refer to the 90% prediction interval.

Figure S6: Diagram of our fitted lung PK profile of ethambutol in mice utilizing the empirical blood PK model by Chen *et al*. as well as the mean lung concentrations of ethambutol after a single oral dose of 100 mg/kg in mice.^29,40^ The blue line represents the fitted healthy/uninvolved lung PK profile of mice, while the blue dots in the Figure are the observed lung concentrations of healthy/uninvolved lung over time.

**Pyrazinamide**

For the extrapolation of pyrazinamide, initially, an empirical model from the literature was utilized to describe the plasma PK of the drug in BALB/c mice after an oral administration of 150 mg/kg which is the human equivalent dose in mice. ^13,43^ It is noted that since BALB/c mice develop only cellular lesions and not caseous lesions, we utilized the literature small cellular lesion PK profiles in TB-patients to validate the extrapolation in humans. ^13^ The observed versus predicted tissue concentrations of the drug are depicted in Figure S7.

Figure S7: Observed versus predicted concentrations of pyrazinamide in BALB/c mice, after a single oral dose of 150 mg/kg, in lung (blue dots) as well as in cellular lesion (green dots). ^13^ The black solid line represents the unity line, while the dashed lines refer to the 90% prediction interval.

**Isoniazid**

Regarding isoniazid, initially, a literature empirical model was utilized to describe the blood PK in mice after a single oral administration (5 mg/kg). ^40^This oral dose was chosen taking into consideration the human equivalent dose of isoniazid in mice. More particularly, the dose of 25 mg/kg in mice, which considered the human equivalent dose, produces a Cmax value much greater than the respective in humans after an oral administration of 5 mg/kg and an AUC_0h-24h_ value similar to the one observed in slow acetylating humans. On the other hand, the dose of 6.25 mg/kg in mice, produces a Cmax value similar to the one observed in humans after a 5 mg/kg dose. However, the obtained AUC_0h-24h_ value in mice, after a dose of 6.25 mg/kg, is much lower than the respective AUC observed in slow acetylating humans, but approximately the same in rapid acetylators. ^45^

In the human study utilized for isoniazid (oral administration of 300 mg), the majority of patients were fast acetylators - high metabolizers (11 out of the 15 subjects were fast acetylators, 2 slow and 2 intermediate).^35^ Also, since in mice the only available lung concentrations in the literature to us were those after an oral administration of 5 mg/kg and 25 mg/kg of isoniazid, we considered the oral administration of 5 mg/kg in mice as the most appropriate in terms of human equivalence and hence utilized it to extrapolate to humans.^29^ However, we acknowledge that an oral dose of 10 mg/kg in mice would be the best option for the extrapolation of lung PK to a typical mean human, according to more recent literature. ^46,47^ The final fitting of the drug’s lung PK in mice is depicted in Figure S8.

Figure S8: Diagram of fitted lung PK profile in mice after an oral administration of isoniazid of 5 mg/kg utilizing the empirical blood PK model by Chen *et al*. as well as the mean lung concentrations of the drug. ^29,40^ The blue line represents the fitted healthy/uninvolved lung PK profile in mice, while the blue dots in the Figure are the mean observed lung concentrations.

# Section 6: Extrapolation of lung PK profiles from preclinical species to humans

In this section of the supplementary material, the results of the extrapolation of the PBPK models from preclinical species to humans are reported as well as their comparisons to literature data. These are reported per compound studied, except for rifampicin where the respective results appear in the main part of the article.

**Moxifloxacin**

The extrapolated lung PK profile of moxifloxacin in humans from rabbits is shown in Figure S9.

Figure S9: Diagram of the extrapolated lung PK profile of moxifloxacin in humans from rabbits. The dashed red line represents the literature plasma PK of a typical mean TB-patient after an oral dose of 400 mg of Moxifloxacin, while the dashed blue, green and brown lines represent the empirical literature healthy/uninvolved lung (Figure A), cavity wall (Figure B) and cavity caseum (Figure C) PK profiles of humans respectively. ^35^The solid light blue, green and brown lines depict our extrapolated lung PBPK profiles of healthy lung, cellular lesion and cavity caseum respectively in TB-patients.

Regarding the extrapolation of moxifloxacin from rabbits to humans, sensitivity analysis was performed for the permeability value of the capillaries. As depicted in Figure S10, the model was sensitive to the capillary permeability, affecting not only lung PK, but also cellular lesion and caseum PK profiles. Obviously, the limiting step concerning the rate of distribution of moxifloxacin within the whole lung (to which the observed data refer to) will be determined by the lower permeability value, which is in this case is the capillary (optimized value = 6.0 cm/h) and not the cellular (optimized value = 9.9 cm/h) permeability. It is highlighted that the simulation with the lower capillary permeability value, compared to the optimized one in rabbits, produced better extrapolation results, especially for lung and caseum PK profiles (Figure S10-A). This could be attributed to the lower abundance of P-gp in the luminal surface area of pulmonary endothelial cells in humans compared to rabbits. In our model only the difference in surface area has been considered between species due to lack of literature data of P-gp abundance in rabbits and humans. However, relevant *in vitro* permeability data are essential to confirm this hypothesis.

Figure S10: Sensitivity analysis of the capillary permeability value to investigate its impact on the tissue PK profiles of moxifloxacin after an oral dose of 400 mg in humans. The dashed blue, green and brown lines represent the empirical literature healthy/uninvolved lung, cavity wall and cavity caseum PK profiles of humans respectively. The solid light blue, green and brown lines depict our extrapolated lung PBPK profiles of healthy lung, cellular lesion and cavity caseum respectively in TB-patients. In Figure A the permeability of the capillaries was assumed 1 cm/h, in Figure B it was assumed 6.0 cm/h (same as the optimized values in rabbits) and in Figure C it was assumed 10 cm/h. In all cases, the cellular permeability was equal to the optimized value in rabbits (9.9 cm/h).

**Ethambutol**

In order to derive the empirical plasma PK of ethambutol in humans, needed to drive the lung PK, we digitized and utilized the mean raw serum concentrations after an oral administration of 25 mg/kg of the drug in healthy fasted subjects. ^44^The empirical model was developed in Monolix 2021R1 and was a two-compartment model with first order absorption and a constant error model. The final parameter estimates for this model and the individual fit of the model to the mean observed serum concentrations of the drug in fasted humans are depicted in Table S11 and Figure S11, respectively. The extrapolated lung PK profile of ethambutol in humans from rabbits and mice is shown in Figure S12.

Table S11: plasma PK parameter values of ethambutol in humans obtained with Monolix utilizing the mean data of 14 fasted healthy subjects.

| PARAMETER | VALUE | sTANDARD ERROR | RELATIVE STANDARD ERROR (%) |
| --- | --- | --- | --- |
| Fixed Effects | | | |
| Tlag (h) | 0.38 | 0.031 | 8.25 |
| Ka (1/h) | 0.51 | 0.12 | 23.7 |
| Cl (L/h) | 59.52 | 14.57 | 24.5 |
| V1 (L) | 214.62 | 50.64 | 23.6 |
| Q (L/h) | 44.22 | 13.82 | 31.2 |
| V2 (L) | 739.1 | 618.45 | 83.7 |
| Error Model Parameters | | | |
| a (mg/L) | 0.16 | 0.022 | 14.1 |

Abbreviations: Tlag, finite time taken for ethambutol to appear in systemic circulation; Ka, absorption rate constant; Cl, clearance of elimination; Q, inter-compartmental clearance; V1, volume of distribution of first compartment; V2, volume of distribution of second compartment; a, constant error term

Figure S11: Diagram of our fitted serum PK profile of ethambutol in humans utilizing the mean raw serum concentrations after an oral administration of 25 mg/kg of the drug in healthy fasted subjects by Peloquin *et al*. ^44^The red line represents the fitted serum PK profile, while the red dots in the Figure are the observed serum concentrations.

Figure S12: Diagram of our extrapolated lung PK profile of ethambutol in humans from rabbits (Figure A) and mice (Figure B). The red line represents the plasma PK of a typical mean healthy individual after an oral dose of 15 mg/kg of Ethambutol, while the blue line represents our extrapolated intracellular lung PK in humans. The red and blue dots represent the actual mean raw serum and alveolar lung cell concentrations respectively.^42^

**Pyrazinamide**

The extrapolated lung PK profile of pyrazinamide in humans from mice is shown in Figure S13.

Figure S13: Diagrams of our extrapolated healthy/uninvolved lung (Figure A) and cellular lesion (Figure B) PK profile of pyrazinamide in humans from mice. The dashed red line represents the plasma PK of a typical mean TB-patient after an oral dose of 1500 mg of pyrazinamide. The dashed blue and green lines represent the empirical literature healthy/uninvolved lung and cellular lesion PK profiles of humans, respectively. ^35^Also, the solid light blue and green lines depict our extrapolated lung and cellular lesion PBPK profile in TB-patients.

**Isoniazid**

The extrapolated lung PK profile of isoniazid in humans from mice is shown in Figure S14.

Figure S14: Diagram of our extrapolated lung PK profile of isoniazid in humans from mice. The dashed red line represents the plasma PK of a typical mean TB-patient after an oral dose of 300 mg of isoniazid, while the dashed blue line represents the empirical literature healthy/uninvolved lung PK profile of humans. ^35^The solid light blue line depicts our extrapolated lung PBPK profile in TB-patients.

# Section 7: Predicted Unbound Fractions

Table S12. Predicted fraction unbound in EW, IW and cellular lesion for rifampicin, moxifloxacin, ethambutol, pyrazinamide and isoniazid in humans

| **Drug** | **Parameter** | **Value** | **Comments** |
| --- | --- | --- | --- |
| Rifampicin | fu_EW_ | 1 | It was assumed 1 |
|  | fu_IW_ | 0.058  (from rabbits)  0.044  (from mice) | It was predicted based on equations (5) and (6) |
|  | fu_Cellular lesion_ | 0.174 | It was predicted based on equation (14) |
| Moxifloxacin | fu_EW_ | 1 | It was assumed 1 |
|  | fu_IW_ | 0.191 | It was predicted based on equations (5) and (6) |
|  | fu_Cellular lesion_ | 0.757 | It was predicted based on equation (14) |
| Ethambutol | fu_EW_ | 1 | It was assumed 1 |
|  | fu_IW_ | 0.25  (from rabbits)  0.24  (from mice) | It was predicted based on equations (5) and (6) |
| Pyrazinamide | fu_EW_ | 0.81 | It was predicted based on equations (8) and (9) |
|  | fu_IW_ | 0.98 | It was predicted assuming that the affinity constant for the acidic phospholipids in lung is 0. |
|  | fu_Cellular lesion_ | 0.991 | It was predicted based on equation (14) |
| Isoniazid | fu_EW_ | 0.64 | It was predicted based on equations (8) and (9) |
|  | fu_IW_ | 0.984 | It was predicted assuming that the affinity constant for the acidic phospholipids in lung is 0. |

# References

1. Rodgers T, Leahy D, Rowland M. Physiologically-based pharmacokinetic modelling 1: Predicting the tissue distribution of moderate-to-strong bases. Journal of Pharmaceutical Sciences 2005; **94**: 1259-1276. Errata: *Journal of Pharmaceutical Sciences* 2007; **96**: 3151-3152.
2. Rodgers T and Rowland M. Physiologically-based pharmacokinetic modelling 2: predicting the tissue distribution of acids, very weak bases, neutrals and zwitterions. *Journal of Pharmaceutical Sciences* 2006; **95**: 1238-1257. Errata: *Journal of Pharmaceutical Sciences* 2007; **96**: 3153-3154.
3. Rodgers T and Rowland M. Mechanistic approaches to volume of distribution predictions: understanding the processes. *Pharmaceutical Research* 2007; **24**: 918-933.
4. Jamei M, Bajot F, Neuhoff S *et al.* A mechanistic framework for in vitro-in vivo extrapolation of liver membrane transporters: prediction of drug-drug interaction between rosuvastatin and cyclosporine. *Clin Pharmacokinet.* 2014; **53**:73-87.
5. Berezhkovskiy LM. A valid equation for the well-stirred perfusion limited physiologically based pharmacokinetic model that consistently accounts for the blood-tissue drug distribution in the organ and the corresponding valid equation for the steady state volume of distribution. *J Pharm Sci.* 2009; **99**: 475–485.
6. Litjens CHC, Verscheijden LFM, Bolwerk C *et al.* Prediction of Moxifloxacin Concentrations in Tuberculosis Patient Populations by Physiologically Based Pharmacokinetic Modeling. *J Clin Pharmacol*. 2022; **62**: 385-396.
7. Olsson B, Bondesson E., Borgström L. *et al.* Controlled Pulmonary Drug Delivery. In: Smyth, H. & Hickey, A., ed. *Pulmonary drug metabolism, clearance, and absorption.* Springer, 2011; 21–50.
8. Johnson DA, Smith KD. The efficacy of certain anti-tuberculosis drugs is affected by binding to alpha-1-acid glycoprotein. *Biomed Chromatogr.* 2006; **20**: 551-560.
9. Ungewiss J, Gericke S, Boriss H. Determination of the Plasma Protein Binding of Liraglutide Using the Escalate* Equilibrium Shift Assay. *J Pharm Sci.* 2019; **108**: 1309-1314.
10. Kempker RR, Heinrichs MT, Nikolaishvili K *et al.* Lung Tissue Concentrations of Pyrazinamide among Patients with Drug-Resistant Pulmonary Tuberculosis. *Antimicrob Agents Chemother.* 2017; **61**: e00226-17.
11. Pienaar E, Sarathy J, Prideaux B *et al.* Comparing efficacies of moxifloxacin, levofloxacin and gatifloxacin in tuberculosis granulomas using a multi-scale systems pharmacology approach. *PloS Comput Biol.* 2017; **13**: e1005650.
12. Mor N, Simon B, Mezo N, Heifets L. Comparison of activities of rifapentine and rifampin against Mycobacterium tuberculosis residing in human macrophages. *Antimicrob Agents Chemother*. 1995; **39**: 2073-2077.
13. Irwin SM, Prideaux B, Lyon ER *et al.* Bedaquiline and Pyrazinamide Treatment Responses Are Affected by Pulmonary Lesion Heterogeneity in Mycobacterium tuberculosis Infected C3HeB/FeJ Mice. *ACS Infect Dis.* 2016; **2**: 251-267.
14. Sarathy JP, Zuccotto F, Hsinpin H *et al.* Prediction of Drug Penetration in Tuberculosis Lesions. *ACS Infect Dis.* 2016; **2**: 552-63.
15. Kaya F, Ernest JP, LoMauro K *et al.* A Rabbit Model to Study Antibiotic Penetration at the Site of Infection for Nontuberculous Mycobacterial Lung Disease: Macrolide Case Study. *Antimicrob Agents Chemother.* 2022; **66**: e0221221.
16. Brillault, J., De Castro, W. V., Harnois *et al.* P-glycoprotein-mediated transport of moxifloxacin in a Calu-3 lung epithelial cell model. *Antimicrobial agents and chemotherapy*. 2009; **53**: 1457–1462.
17. Urbanowski ME, Ordonez AA, Ruiz-Bedoya CA *et al.* Cavitary tuberculosis: the gateway of disease transmission. *Lancet Infect Dis.* 2020; **20**: e117-e128.
18. Simcyp™ PBPK Simulator. *The Standard for Population-based Physiologically Based Modeling and Simulation Updated with Version 22 Capabilities*; 2023 [https://www.certara.com/software/simcyp-pbpk/](https://www.certara.com/software/simcyp-pbpk/))
19. Gill KL, Gardner I, Li L, Jamei M. A Bottom-Up Whole-Body Physiologically Based Pharmacokinetic Model to Mechanistically Predict Tissue Distribution and the Rate of Subcutaneous Absorption of Therapeutic Proteins. *AAPS J.* 2016; **18**: 156-70.
20. Gehr P, Mwangi DK, Ammann A *et al.* Design of the mammalian respiratory system. V. Scaling morphometric pulmonary diffusing capacity to body mass: wild and domestic mammals. *Respir Physiol.* 1981; **44**: 61-86.
21. Stone KC, Mercer RR, Gehr P *et al.* Allometric relationships of cell numbers and size in the mammalian lung. *Am J Respir Cell Mol Biol.* 1992; **6**: 235-43.
22. Iversen NK, Malte H, Baatrup E *et al.* The normal acid-base status of mice. *Respir Physiol Neurobiol.* 2012; **180**: 252-7.
23. GastroPlus® PBBM / PBPK. *Modeling and simulation package – supporting internal research through regulatory filings;* 2023

<https://www.simulations-plus.com/software/gastroplus/>

1. Heming TA, Bidani A. Effects of plasmalemmal V-ATPase activity on plasma membrane potential of resident alveolar macrophages. *Lung.* 2003; **181**: 121-35.
2. Gaohua L, Wedagedera J, Small BG *et al.* Development of a Multicompartment Permeability-Limited Lung PBPK Model and Its Application in Predicting Pulmonary Pharmacokinetics of Antituberculosis Drugs. *CPT Pharmacometrics Syst Pharmacol.* 2015; **4**: 605-13.
3. Ruark CD, Hack CE, Robinson PJ *et al.* Predicting passive and active tissue:plasma partition coefficients: interindividual and interspecies variability. J Pharm Sci. 2014; **103**: 2189-2198.
4. National Center for Biotechnology Information. *PubChem Compound Summary for CID 135398735, Rifampin.* 2024

<https://pubchem.ncbi.nlm.nih.gov/compound/Rifampin>.

1. Humphries H, Almond L, Berg A *et al.* Development of physiologically-based pharmacokinetic models for standard of care and newer tuberculosis drugs*. CPT Pharmacometrics Syst Pharmacol.* 2021; **10**: 1382-1395.
2. Muliaditan M, Teutonico D, Ortega-Muro F *et al.* Prediction of lung exposure to anti-tubercular drugs using plasma pharmacokinetic data: Implications for dose selection. *Eur J Pharm Sci.* 2022; **173**: 106163.
3. Rowland M and Tozer T *Clinical Pharmacokinetics, Concepts and Applications*. Lippincott Williams & Wilkins. 1995
4. Davies B and Morris T Physiological parameters in laboratory animals and humans. *Pharm Res.* 1993; **10**: 1093–1095.
5. National Center for Biotechnology Information. *PubChem Compound Summary for CID 14052, Ethambutol*. 2024

<https://pubchem.ncbi.nlm.nih.gov/compound/Ethambutol>.

1. Edginton AN, Ahr G, Willmann S *et al.* Defining the role of macrophages in local moxifloxacin tissue concentrations using biopsy data and whole-body physiologically based pharmacokinetic modelling. *Clin Pharmacokinet.* 2009; **48**: 181-187.
2. National Center for Biotechnology Information. *PubChem Compound Summary for CID 3767, Isoniazid.* 2024

<https://pubchem.ncbi.nlm.nih.gov/compound/Isoniazid>.

1. Strydom N, Gupta SV, Fox WS *et al.* Tuberculosis drugs’ distribution and emergence of resistance in patient’s lung lesions: A mechanistic model and tool for regimen and dose optimization. *PloS Med.* 2019; **16**: e1002773.
2. National Center for Biotechnology Information. *PubChem Compound Summary for CID 1046, Pyrazinamide.* 2024

<https://pubchem.ncbi.nlm.nih.gov/compound/Pyrazinamide>.

1. Sarathy J, Blanc L, Alvarez-Cabrera N *et al.* Fluoroquinolone Efficacy against Tuberculosis Is Driven by Penetration into Lesions and Activity against Resident Bacterial Populations. *Antimicrob Agents Chemother.* 2019; **63**: e02516-18.
2. Rifat D, Prideaux B, Savic RM *et al.* Pharmacokinetics of rifapentine and rifampin in a rabbit model of tuberculosis and correlation with clinical trial data. *Sci Transl Med.* 2018; **10**: eaai7786.
3. Zimmerman M, Lestner J, Prideaux B *et al.* Ethambutol Partitioning in Tuberculous Pulmonary Lesions Explains Its Clinical Efficacy. *Antimicrobic Agents Chemother.* 2017; **61:** e00924-17.
4. Chen C, Ortega F, Alameda L *et al.* Population pharmacokinetics, optimized design and sample size determination for rifampicin, isoniazid, ethambutol and pyrazinamide in the mouse. *Eur J Pharm Sci.* 2016; **93**: 319-33.
5. Blanc L, Daudelin IB, Podell BK *et al.* High-resolution mapping of fluoroquinolones in TB rabbit lesions reveals specific distribution in immune cell types. *Elife.* 2018; **7**: e41115.
6. Conte JE Jr, Golden JA, Kipps J *et al.* Effects of AIDS and gender on steady-state plasma and intrapulmonary ethambutol concentrations. *Antimicrob Agents Chemother.* 2001; **45**: 2891-6.
7. Chen C, Wicha SG, de Knegt GJ *et al.* Assessing Pharmacodynamic Interactions in Mice Using the Multistate Tuberculosis Pharmacometric and General Pharmacodynamic Interaction Models. *CPT Pharmacometrics Syst Pharmacol.* 2017; **6**: 787-797.
8. Peloquin CA, Bulpitt AE, Jaresko GS *et al.* Pharmacokinetics of ethambutol under fasting conditions, with food, and with antacids. *Antimicrob Agents Chemother.* 1999; **43**: 568-572.
9. Almeida D, Nuermberger E, Tasneen R *et al.* Paradoxical effect of isoniazid on the activity of rifampin-pyrazinamide combination in a mouse model of tuberculosis. *Antimicrob Agents Chemother.* 2009; **53**: 4178-84.
10. Dutta NK, Pinn ML, Karakousis PC. Reduced emergence of isoniazid resistance with concurrent use of thioridazine against acute murine tuberculosis. *Antimicrob Agents Chemother.* 2014; **58**: 4048-53.
11. Kaushik A, Ammerman NC, Tasneen R *et al.* Efficacy of Long-Acting Bedaquiline Regimens in a Mouse Model of Tuberculosis Preventive Therapy. *Am J Respir Crit Care Med.* 2022; **205**: 570-579.
